# Five Decades of Infusion Testing in Idiopathic Normal-Pressure Hydrocephalus: Resistance to CSF Outflow as a Biomarker in Search of a Gold Standard

**DOI:** 10.3390/biomedicines14102233

**Published:** 2026-10-02

**Authors:** Juan Sahuquillo, Murad Al-Nusaif, Monica Torrecilla-Vall-llossera, Turgut Durduran, Maria-A. Poca

**Affiliations:** 1 Neurotraumatology and Neurosurgery Research Unit, Vall d’Hebron Institut de Recerca (VHIR), Passeig Vall d’Hebron 119-129, 08035 Barcelona, Spain; murad.alnusaif@icfo.eu; 2 ICFO-Institut de Ciències Fotòniques, 08860 Castelldefels, Spain; monica.torrecilla@icfo.eu (M.T.-V.-l.); turgut.durduran@icfo.eu (T.D.); 3 Department of Surgery, Vall d’Hebron Barcelona Hospital Campus, Universitat Autònoma de Barcelona, 08035 Barcelona, Spain; 4 ICREA—Institució Catalana de Recerca i Estudis Avançats, 08010 Barcelona, Spain; 5 Department of Neurosurgery, Vall d’Hebron Barcelona Hospital Campus, Vall d’Hebron Hospital Universitari, Passeig de la Vall d’Hebron, 119-129, 08035 Barcelona, Spain

**Keywords:** idiopathic normal-pressure hydrocephalus, lumbar infusion test, resistance to CSF outflow, diagnostic biomarker, Bayesian analysis, likelihood ratio, reference standard, shunt responsiveness, CSF proteomics

## Abstract

Idiopathic normal-pressure hydrocephalus (iNPH) is a potentially reversible cause of gait impairment, cognitive decline, and urinary dysfunction in older adults, yet identifying patients most likely to benefit from cerebrospinal fluid (CSF) diversion remains a major diagnostic challenge. For more than five decades, the lumbar infusion test, introduced by Katzman and Hussey in 1970, has served as the principal method for assessing CSF absorptive capacity through measurement of resistance to CSF outflow (*R*_out_). This narrative review examines the historical development, physiological basis, and contemporary clinical application of infusion testing in iNPH, with particular emphasis on the diagnostic and prognostic value of *R*_out_. We critically review the evidence supporting the *R*_out_ thresholds recommended by current international, Japanese, and American Academy of Neurology guidelines and reassess their performance using a Bayesian framework. Recalculation of likelihood ratios from published meta-analyses demonstrates that none of the commonly proposed *R*_out_ thresholds (10–18 mmHg·min/mL) achieves the prognostic performance typically associated with a strong rule-in or rule-out test. We argue that this limited performance reflects not only methodological heterogeneity in infusion-testing protocols but also a more fundamental problem: the absence of a universally accepted reference standard for iNPH. We further examine the limitations of using postoperative clinical improvement as a surrogate reference standard, including variability in outcome definitions, delayed treatment effects, and the potential for circular reasoning. Finally, we review emerging CSF proteomic biomarkers of neuroinflammation, axonal injury, and concomitant Alzheimer-type pathology that may complement hydrodynamic assessment by capturing the balance between reversible hydrocephalus-related dysfunction and irreversible neurodegenerative burden. We conclude that *R*_out_ should be regarded as a valuable but incomplete biomarker and that future progress will likely depend on multimodal, probabilistically interpreted models integrating CSF hydrodynamics, neuroimaging, and molecular biomarkers to improve prognostic stratification and establish more robust diagnostic frameworks for iNPH.

## 1. Introduction

In 1964, Salomón Hakim described a 16-year-old patient who, following surgical evacuation of an acute subdural hematoma, remained in what would now be considered a “minimally conscious state” [1]. Bilateral carotid arteriography demonstrated hydrocephalus, and lumbar puncture revealed a cerebrospinal fluid (CSF) opening pressure of 150 mmH_2_O [1]. Remarkably, the patient showed clear clinical improvement within 24 h of the removal of 15 mL of CSF, including recovery of the ability to follow commands. This improvement was followed by a dramatic clinical response after ventriculoatrial shunt (VAS) placement [1]. In the same 1964 report, Hakim described two additional patients. The second case involved a 52-year-old man who was admitted for evaluation of progressive cognitive impairment, gait instability, and urinary incontinence, with an initial clinical suspicion of a neurodegenerative disorder. Neuroimaging revealed ventriculomegaly, and treatment with a VAS resulted in marked clinical improvement, with recovery to an almost normal functional state [1]. A third case involved a patient with post-traumatic hydrocephalus who also demonstrated partial clinical recovery following surgery. The paradoxical and unexpected observation that patients with symptomatic hydrocephalus may exhibit normal intracranial pressure (ICP), yet experience substantial clinical improvement following shunt surgery, prompted early efforts to elucidate the pathophysiology of what is now known as normal-pressure hydrocephalus (NPH). Hakim further proposed that ventriculomegaly associated with a dementia-like syndrome was not necessarily irreversible and that selected patients could achieve meaningful clinical improvement after CSF diversion [1].

Building on Hakim’s initial report, Hakim and Adams published a second paper in 1965 that revisited two of the cases originally described in 1964 and summarized their experience with a “*larger series of cases observed over the previous 10 years*” [2]. In this work, the authors also introduced the concept of the “hydraulic press” as a mechanistic explanation for hydrocephalus despite normal ICP. They proposed that ventricular enlargement increases the surface area over which CSF pressure acts, allowing relatively normal ICP to generate abnormal distending forces on the ventricular walls and adjacent brain tissue [2]. This conceptual framework was later expanded in the influential paper by Adams et al. published in the *New England Journal of Medicine*, which firmly established and broadened the clinical and pathophysiological concept of NPH [3]. Together, these three papers, coupled with the concluding statement by Adams et al.—that “*recognition and treatment could effectively ‘cure’ a condition resembling presenile or senile dementia”* [3]—signaled a paradigm shift in neurosurgery. Nevertheless, as with many transformative concepts, the emergence of NPH was accompanied by considerable controversy, and despite decades of research, several key questions regarding its pathophysiology, diagnosis, and management remain unresolved and continue to generate debate.

Idiopathic normal-pressure hydrocephalus (iNPH) is characterized by a complete or incomplete clinical triad of gait impairment, cognitive impairment, and urinary urgency or incontinence, together with ventriculomegaly on neuroimaging, after exclusion of secondary causes of hydrocephalus [4,5]. Despite continued reluctance and skepticism among some neurologists [6,7], iNPH remains a potentially reversible neurological syndrome when patients are appropriately selected for CSF diversion and treated with modern shunt systems that incorporate technical advances introduced over the past two decades, particularly gravitational control mechanisms [5,8,9]. While ventriculoperitoneal shunting remains the predominant intervention in Western countries, lumboperitoneal shunting has gained widespread adoption in East Asian countries, particularly Japan, where it accounts for more than half of shunting procedures performed for iNPH. Two recent systematic reviews have demonstrated comparable clinical outcomes following ventriculoperitoneal and lumboperitoneal shunting, with no significant differences in overall clinical improvement or gait recovery [9,10]. In their systematic review, Palermo et al. concluded that neither procedure is clearly superior, emphasizing that treatment decisions should be individualized according to patient characteristics, anatomical considerations, and surgical risk factors [10].

In some patients, CSF shunting results in only partial improvement of gait and urinary symptoms, whereas others demonstrate marked functional recovery. Still others fail to improve despite apparently appropriate patient selection and technically successful shunt placement. At the same time, growing evidence suggests that iNPH commonly coexists with other neurodegenerative disorders, including Alzheimer’s disease, Parkinson’s disease, vascular dementia, and progressive supranuclear palsy [5,11,12]. Such comorbidities may alter the clinical presentation, complicate diagnostic assessment, and contribute substantially to the variability in postoperative outcomes observed after surgery.

The exact prevalence of iNPH remains difficult to establish because reported estimates vary substantially according to the diagnostic criteria applied and the populations studied. Despite this uncertainty, iNPH is considered the most common form of hydrocephalus in individuals aged 65 years and older [13]. In a population-based Swedish study, Andersson et al. (2019) reported a prevalence of probable iNPH of 3.7% among individuals aged ≥ 65 years, rising to 8.9% among those aged 80 years and older [14]. According to the Global Burden of Disease Study 2021, life expectancy increased by 22.7 years worldwide between 1950 and 2021 [15]. As populations age and the prevalence of both neurodegenerative disorders and ventriculomegaly increases, distinguishing iNPH from hydrocephalus *ex vacuo* and incidental ventricular enlargement becomes increasingly important. The 2021 Japanese guidelines, now widely adopted internationally, classify patients as having possible, probable, or unlikely iNPH. However, even a diagnosis of probable iNPH does not reliably predict a favorable response to shunt surgery [4]. A key challenge is to identify patients in whom ventricular enlargement primarily reflects potentially reversible CSF-dynamic disturbances rather than underlying neurodegenerative pathology, which is generally associated with a lower likelihood of responding to CSF diversion. Failure to make this distinction may expose a vulnerable elderly population to unnecessary surgical risks while limiting the potential benefits of treatment. Reported improvement rates following CSF diversion in patients with probable iNPH vary considerably across studies, ranging from approximately 40% in earlier, more heterogeneous cohorts [16] to more than 75% in recent series using rigorous patient-selection criteria [5,17]. This variability likely reflects differences in patient selection, outcome measures, follow-up duration, and the biological and clinical heterogeneity of the iNPH population. Importantly, it should not be interpreted as evidence of limited efficacy of shunt surgery. Rather, treatment response exists along a continuum and is influenced by multiple factors, including baseline clinical status, comorbidities, delays in diagnosis and treatment, and the coexistence of neurodegenerative pathology. Consequently, the primary goal in patients with suspected iNPH is not merely to establish the diagnosis, but to identify those most likely to benefit from CSF diversion. Ancillary investigations therefore play a crucial role in bridging the gap between clinical suspicion and treatment decisions.

Current guidelines recommend several ancillary investigations for iNPH, including the CSF tap test, external lumbar drainage, ICP monitoring, and the lumbar infusion test (LIT). Although each provides valuable information, all are imperfect and differ in invasiveness, technical requirements, diagnostic performance, and prognostic value. Among these, the constant-rate LIT, first described by Katzman and Hussey in 1970 and subsequently refined over five decades, occupies a prominent role in the evaluation of suspected iNPH [18]. Typically performed as an outpatient or short-stay procedure, it enables quantification of *R*_out_, a parameter closely linked to iNPH pathophysiology. The LIT is endorsed by most clinical practice guidelines, and its diagnostic and prognostic performance has been extensively evaluated in large single-center and multicenter cohorts. Nevertheless, considerable uncertainty remains regarding the optimal *R*_out_ threshold for predicting shunt responsiveness across different patient populations [4,19,20,21].

The primary aim of this review is to critically evaluate the diagnostic and prognostic value of the LIT in patients with iNPH, with particular emphasis on resistance to CSF outflow (*R*_out_) as its principal biomarker. To achieve this objective, we trace the evolution of infusion testing from the pioneering studies of the 1970s to contemporary computerized assessments of CSF dynamics and examine the physiological foundations and methodological challenges that continue to complicate its interpretation. We critically assess the evidence supporting guideline-recommended *R*_out_ thresholds and evaluate *R*_out_ as both a diagnostic and prognostic biomarker, highlighting persistent uncertainty regarding optimal cutoff values, variability in predictive performance, and the challenge of identifying patients most likely to benefit from CSF diversion. We also examine a fundamental methodological problem underlying diagnostic studies in iNPH: the absence of a universally accepted reference standard. Using a Bayesian framework, we explore how this limitation influences the interpretation of infusion-test results and constrains evaluation of their diagnostic and prognostic value. Finally, we place infusion testing within its broader physiological context by reviewing CSF production, circulation, and absorption, and discuss how emerging molecular biomarkers, machine-learning approaches, and multimodal predictive models may complement hydrodynamic measurements in future diagnostic strategies.

## 2. Methods and Sources of Evidence

This article was conceived as a narrative review of the historical development, physiological foundations, and clinical application of the LIT in iNPH. The primary objectives of this review were threefold: (1) to critically evaluate the diagnostic and prognostic value of the lumbar infusion test (LIT) in patients with iNPH, with particular emphasis on resistance to CSF outflow (*R*_out_); (2) to reassess its clinical utility within a Bayesian framework; and (3) to examine how the absence of a universally accepted reference standard influences the interpretation of diagnostic studies and the assessment of treatment responsiveness in iNPH.

Relevant publications published through 25 May 2026 were identified through searches of PubMed, Scopus, and Web of Science using combinations of keywords related to idiopathic normal-pressure hydrocephalus, CSF dynamics, lumbar infusion testing, resistance to CSF outflow, CSF tap testing, external lumbar drainage, ICP monitoring, neuroimaging, biomarkers, and shunt responsiveness. The search strategy was supplemented by backward and forward citation tracking of key articles, review of major clinical practice guidelines for iNPH, and literature mapping using Research Rabbit and Connected Papers to identify influential studies and citation networks [22,23]. Studies were considered eligible if they addressed the diagnosis, pathophysiology, CSF hydrodynamics, infusion testing, shunt responsiveness, or biomarker research in iNPH. Particular emphasis was placed on studies addressing CSF dynamics, infusion testing, *R*_out_ diagnostic accuracy, shunt responsiveness, and emerging biomarkers in iNPH.

### 2.1. Selection of Studies Evaluating the Predictive Performance of the LIT

For the assessment of *R*_out_ as a diagnostic and prognostic biomarker, priority was given to studies incorporated into the International Guidelines, the Japanese Guidelines for iNPH, and the American Academy of Neurology practice guideline, as these constitute the principal evidence base underpinning contemporary clinical practice [4,19,24]. Additional evidence was obtained from large prospective cohorts and published meta-analyses identified through database searches and citation-network analysis. Studies reporting sensitivity, specificity, predictive values, likelihood ratios, or other measures of diagnostic performance for predefined *R*_out_ thresholds were independently reviewed by the two senior authors (M.-A.P. and J.S.). Disagreements regarding study inclusion, evidentiary priority, or interpretation were resolved by consensus. When overlapping cohorts were identified, priority was given to studies with the largest sample size, longest follow-up, or most complete reporting of diagnostic accuracy outcomes. Given the narrative nature of this review, no formal risk-of-bias assessment or quantitative synthesis was undertaken.

### 2.2. Re-Examining R_out_ Thresholds Using Likelihood Ratios

To facilitate Bayesian interpretation of published diagnostic-performance data, positive and negative likelihood ratios (LR+ and LR−) were recalculated from reported sensitivity and specificity values using standard formulas [25,26,27]. Sensitivity and specificity estimates were extracted from the major clinical practice guidelines, the primary studies cited therein, and published meta-analyses when available. When multiple estimates were available for a given *R*_out_ threshold, preference was given to those derived from guideline-supported studies, large prospective cohorts, or meta-analyses. No pooling of individual studies was performed, and no formal meta-analysis was undertaken. Consequently, the resulting LRs represent secondary calculations based on published data and should not be interpreted as pooled estimates of diagnostic accuracy.

## 3. CSF Outflow: From Classical Models to Emerging Concepts

Current evidence supports the view that CSF is produced predominantly by energy-dependent secretion within the choroid plexus, replacing earlier theories that regarded passive filtration as the principal mechanism of CSF formation [28]. However, despite decades of investigation, the mechanisms governing CSF drainage from the subarachnoid space remain incompletely understood. Multiple CSF outflow pathways have been described, but their relative importance in maintaining CSF homeostasis remains uncertain and continues to be actively debated. Moreover, much of our current understanding of CSF absorption is based on experimental animal models, and the applicability of these observations to human physiology remains a matter of ongoing controversy [29]. In parallel, growing evidence supporting the existence of the so-called glymphatic system—first characterized in rodent models—has challenged traditional views of CSF–interstitial fluid exchange by emphasizing the role of perivascular pathways in the clearance of metabolic waste from the brain [30,31]. Iliff et al. coined the term “glymphatic” to emphasize the dual nature of this pathway: its reliance on glial-mediated fluid transport and its lymphatic-like function in waste clearance [31]. The glymphatic pathway has been proposed as a brain-wide waste clearance system that functionally compensates for the lack of classical lymphatic vessels within the brain. Nevertheless, its quantitative contribution to CSF clearance, its relationship with conventional absorptive pathways, and its modulation by physiological factors such as sleep, arterial pulsatility, and aging remain incompletely characterized. Consequently, its role in human CSF physiology remains uncertain and continues to be the subject of considerable debate.

**Figure 1:**
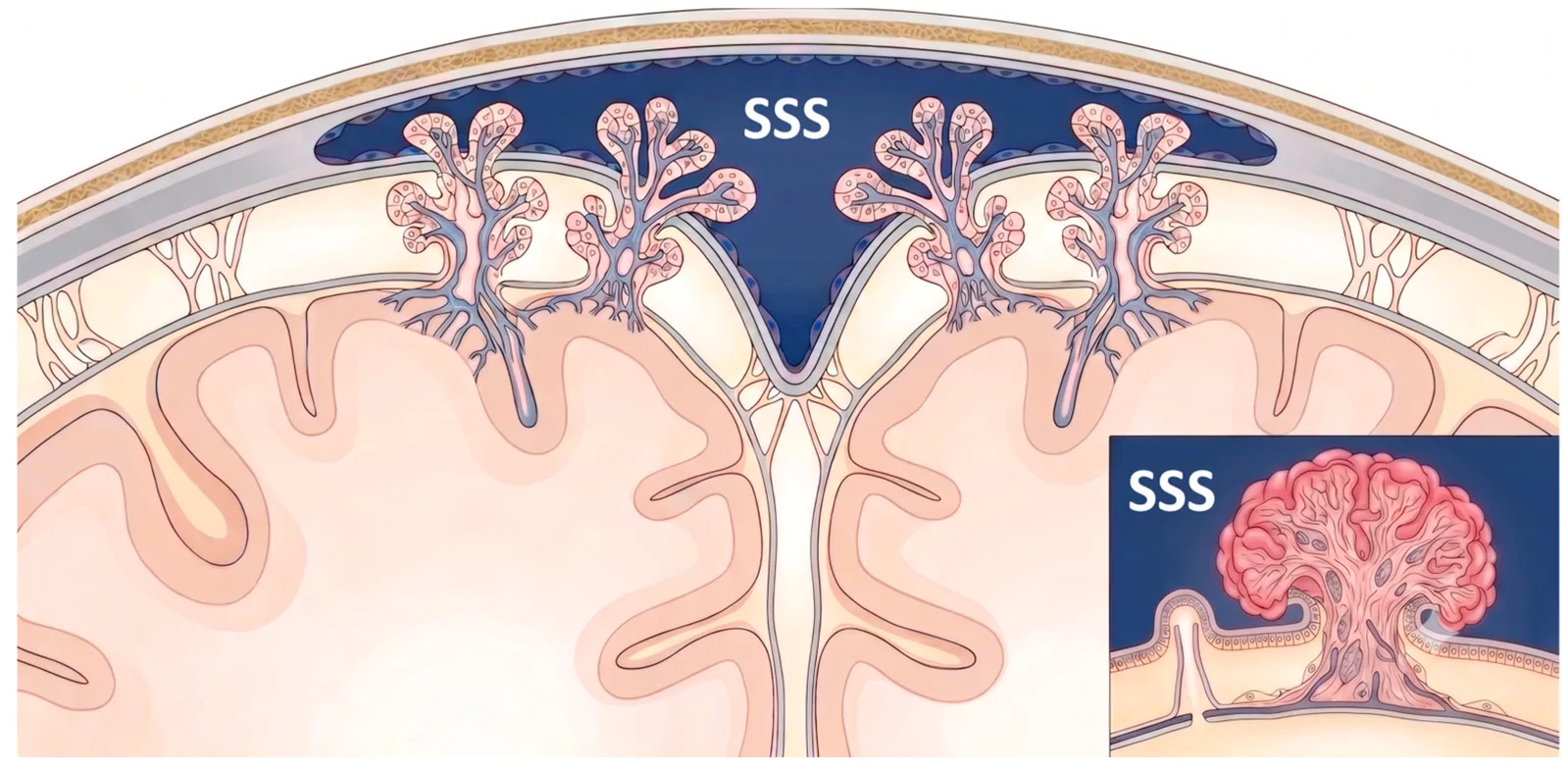
Structural morphology of arachnoid villi and arachnoid (Pacchionian) granulations. Contemporary scientific illustration inspired by the classical observations of Key, Retzius, and Weed, depicting, in coronal section, the anatomical relationships among the cerebral cortex, subarachnoid space, dura mater, arachnoid villi, arachnoid granulations, and the superior sagittal sinus (SSS). The main panel illustrates multiple macroscopic arachnoid granulations projecting through the dura into the lumen of the SSS, consistent with the classical model of cerebrospinal fluid (CSF) absorption. Inset (bottom right): Enlarged schematic illustrating the transition from a simple microscopic arachnoid villus (**left**) to a larger and more complex arachnoid granulation (**right**). Both structures are depicted as specialized protrusions of the arachnoid membrane containing a connective-tissue core and a cellular covering through which CSF has traditionally been thought to pass into the venous circulation. **Abbreviation:** SSS, superior sagittal sinus. Conceptual illustration generated with Gemini (Gemini 1.5 Pro, Google LLC) and reviewed and edited by the authors.

Traditionally, CSF absorption has been attributed primarily to drainage through the arachnoid villi and Pacchionian granulations into the dural venous sinuses, particularly the superior sagittal sinus [32]. More recently, evidence has emerged supporting additional CSF outflow pathways, including meningeal lymphatic and perineural routes. Nevertheless, their quantitative contribution to overall CSF clearance remains uncertain. In their landmark studies published in 1876, Key and Retzius proposed that CSF is absorbed mainly through the arachnoid villi and Pacchionian granulations into the dural venous sinuses [33]. This concept rapidly became the dominant model of CSF absorption and remained so for much of the twentieth century. Using gelatin stained with Prussian blue and injected into the subarachnoid space of cadavers at pressures of approximately 60 mmHg, the investigators demonstrated continuity between the spinal and cranial subarachnoid compartments. The tracer spread throughout the craniospinal subarachnoid space and extended into the cores of the Pacchionian granulations, where it was interpreted as passing across their cellular lining into the dural venous sinuses (Figure 1).

Key and Retzius’ proposal that arachnoid villi and Pacchionian granulations constitute the primary pathway for CSF absorption was subsequently challenged by observations that these structures are absent or only partially developed in newborns, infants, and several higher mammalian species. These findings questioned the universality of the classical absorption model and stimulated the search for alternative pathways involved in CSF drainage and homeostasis [28].

A major body of evidence supporting the model proposed by Key and Retzius was generated by Lewis H. Weed, who, under Harvey Cushing’s mentorship, performed a series of seminal studies in 1914 that helped establish arachnoid villi and Pacchionian granulations as the principal route of CSF absorption [34]. These investigations formed a cornerstone of twentieth-century CSF physiology and strongly influenced Cushing’s concept of the “third circulation” [35].

The concept that arachnoid villi and Pacchionian granulations represent the primary pathway for CSF absorption was subsequently challenged by Dandy and Blackfan, who proposed that CSF absorption occurs more diffusely throughout the subarachnoid space and directly into the vascular circulation [36,37]. They also emphasized the limited development of arachnoid granulations during early life and in several animal species, suggesting the existence of alternative CSF drainage pathways [36,37]. In response to these competing theories, Weed conducted a series of tracer studies that supported the classical view that CSF is absorbed into the venous circulation through specialized arachnoid structures [28,38]. Further support for this model came from Welch and Friedman, who demonstrated in vitro in 1960 that monkey arachnoid villi function as unidirectional valves [39]. Together, these studies established the classical model of CSF circulation that dominated much of the twentieth century and provided the physiological foundation for later infusion tests designed to assess CSF absorptive capacity through measurements of *R*_out_.

## 4. Historical Development of the Katzman Lumbar Infusion Test

**Figure 2:**
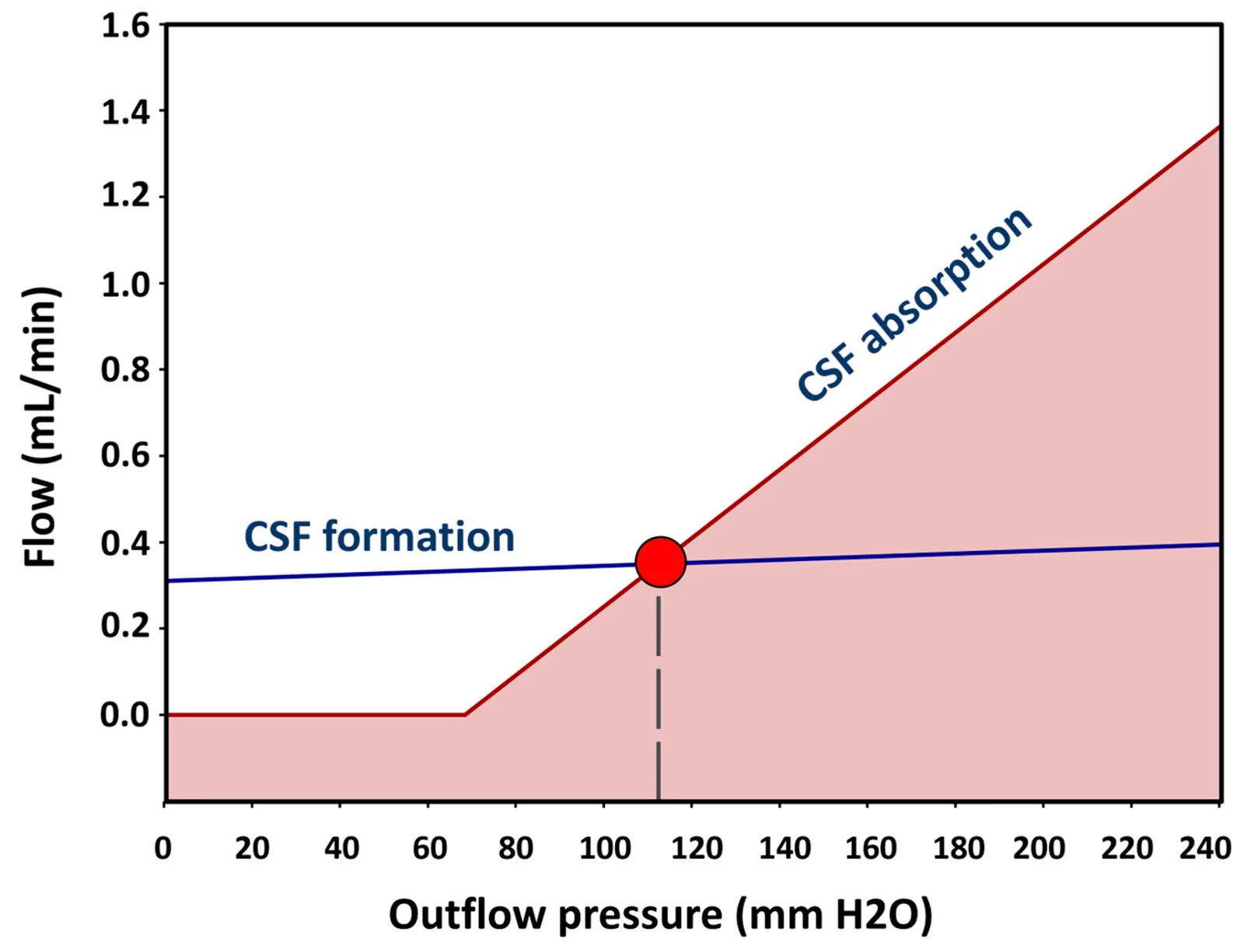
The relationship between cerebrospinal fluid (CSF) flow and outflow pressure, as originally illustrated by Cutler et al. (Cutler et al. Figure 4) [44], is schematically reproduced here using the *ggplot2* package (v3.5.1) in R (v4.6.1) to emphasize key physiological concepts. The figure demonstrates that CSF formation (in blue) remains relatively constant (0.35 mL/min) and is largely independent of intracranial pressure (ICP), whereas CSF absorption (in red) increases linearly once a threshold pressure is exceeded (*P*_0_ ≈ 68 mm H_2_O). At low pressures below this threshold, absorption is zero; beyond it (marked by the red intersection point at *P*_eq_ ≈ 112 mm H_2_O), absorption rises proportionally with increasing pressure. The intersection of the formation and absorption curves defines the equilibrium ICP, at which CSF production equals absorption. This linear pressure–flow relationship provides the physiological basis for the concept of resistance to CSF outflow and underpins modern infusion-based techniques for assessing CSF dynamics.

Although Katzman and Hussey are generally credited with introducing the LIT into clinical practice, the origins of infusion testing can be traced to earlier experimental work. To our knowledge, the first description of a ventriculocisternal infusion technique was published by the Belgian physiologist Isidoor Leusen in 1948. Using canine models, Leusen applied this approach to investigate the influence of CSF ionic composition on vasomotor regulation [40]. The technique was subsequently advanced by Pappenheimer et al. in 1962 through the introduction of inulin—a polysaccharide composed mainly of fructose units—as a tracer, allowing the first quantitative measurements of CSF formation and CSF–blood exchange in goats [41]. In the same year, Rall et al. employed radiolabeled inulin in dogs to estimate the volume of the brain extracellular space [42]. Pappenheimer’s ventriculocisternal infusion technique subsequently became the gold-standard experimental approach for studying CSF physiology and was widely used in animal models to investigate CSF formation, circulation, absorption, and solute exchange. Extending this approach to clinical research, Rubin et al. (1966) used the inulin infusion technique to quantify CSF production and evaluate the effects of acetazolamide in adults with brain tumors [43]. Similarly, Cutler et al. applied the technique in children with panencephalitis or pontine gliomas [44]. Despite differences in patient populations, both studies demonstrated that, within physiological ranges, CSF absorptive capacity is linearly related to ICP [43,44] (Figure 2).

In a landmark study, Lorenzo et al. (1974) applied Pappenheimer’s ventriculolumbar perfusion technique to investigate CSF dynamics in five patients with suspected iNPH [45]. They demonstrated that impaired CSF absorption was the predominant physiological abnormality in all cases and proposed that measuring CSF absorptive capacity could help distinguish iNPH from cerebral atrophy. This study provided some of the earliest clinical evidence linking impaired CSF absorption to the pathophysiology of iNPH and helped establish CSF hydrodynamic testing as a valuable diagnostic tool. Although the Pappenheimer technique was subsequently adopted by a limited number of investigators in both adult and pediatric populations and provided accurate measurements of CSF formation and absorptive capacity, its technical complexity limited its clinical applicability. The need for ventricular cannulation, tracer-based analyses, and prolonged perfusion procedures hindered its widespread adoption and ultimately prompted the development of simpler and more practical methods for assessing CSF dynamics.

### 4.1. Origins and Rationale

The first systematic attempt to investigate CSF dynamics in humans was undertaken by Jules Masserman, a psychiatrist and neurologist [46,47]. In his pioneering 1934 studies, Masserman performed prolonged CSF pressure recordings in patients maintained in the lateral decubitus position for at least two hours and reported a mean ICP of 147.7 mmH_2_O (10.8 mmHg) [47]. He also proposed a simple method for estimating CSF production based on the volume of CSF removed and the time required for ICP to return to baseline. Using this approach, he calculated a mean CSF formation rate of approximately 0.30 mL/min [47]. Several decades later, Rubin et al. (1966) proposed that impaired bulk CSF absorption through the outflow pathways represents the principal pathophysiological mechanism underlying communicating hydrocephalus [43]. In this context, bulk flow refers to the movement of CSF as a fluid mass driven by pressure gradients, in contrast to diffusion, which describes the passive movement of individual molecules along concentration gradients. These concepts laid the foundation for the development of the lumbar infusion test (LIT), which marked a major advance in the clinical application of CSF physiology. The LIT provided a practical bedside method for estimating CSF absorptive capacity without the technical complexity and invasiveness associated with earlier ventriculolumbar perfusion techniques [18].

### 4.2. Foundations of Infusion Testing: The Foldes–Arrowood Studies

The conceptual origins of the Katzman-Hussey test can be traced to the work of Foldes and Arrowood (1948), who investigated the effects of continuous subarachnoid infusion while developing a technique for spinal analgesia using 0.5% intrathecal procaine [48]. In fifteen neurosurgical patients undergoing lumbar puncture, they observed that the infusion of normal saline at rates of 0.2-0.8 mL/min produced a progressive increase in CSF pressure that eventually reached a steady-state plateau [48]. At this point, the rate of infusion was balanced by CSF absorption, providing some of the earliest evidence for the pressure-flow relationship governing CSF dynamics [48]. Infusion rates between 0.30 and 0.65 mL/min typically produced a stable pressure plateau after approximately 40 min, reflecting equilibrium between CSF inflow and absorption [48]. Foldes and Arrowood also observed that cessation of the infusion resulted in a characteristic biphasic decline in CSF pressure, consisting of an initial rapid decrease followed by a slower phase of decay. Although the physiological basis of this pattern was not understood at the time, it later became important for the development of models of intracranial compliance and CSF hydrodynamics. Based on these findings, they concluded that the method could serve as *“a neurological diagnostic procedure that would give quantitative information as to the changes in CSF absorption in various pathological conditions”* [48]. Today, CSF absorptive capacity is commonly quantified by the resistance and conductance to CSF outflow (R_out_ and C_out_), the principal hydrodynamic parameters derived from the lumbar infusion test (LIT).

### 4.3. The Classical Katzman–Hussey Infusion Test

Robert Katzman (1925–2008) was a neurologist whose work profoundly influenced the understanding of Alzheimer’s disease (AD) [49]. In collaboration with Francis Hussey, he developed the constant-infusion manometric test—later termed the lumbar infusion test (LIT)—as a clinically applicable bedside method for quantifying CSF absorptive capacity. Their seminal 1970 report in *Neurology* marked a pivotal transition from experimental CSF hydrodynamics to routine clinical evaluation, providing the foundation for infusion-based assessment of communicating hydrocephalus [18,50].

In the original test, sterile saline was infused into the lumbar subarachnoid space at a rate of 0.76 mL/min, approximately twice the normal rate of CSF formation, while CSF pressure was monitored until a stable plateau was reached, typically within 40-60 min [18]. Katzman and Hussey proposed that the normal CSF absorptive system possesses substantial reserve capacity, allowing absorption rates several times higher than physiological CSF production before saturation occurs [18]. In a subsequent publication, Katzman and Hussey reported findings in 40 patients (9 children and 31 adults), further validating the clinical utility of the technique [50]. Three characteristic pressure-response patterns were identified: a “normal” pattern, with a plateau pressure below 300 mmH_2_O (22 mmHg); a “severely abnormal” pattern, in which pressure exceeded 500 mmH_2_O (36.7 mmHg) within 10 min of infusion; and an “intermediate” pattern characterized by a more gradual increase beyond 300 mmH_2_O, suggesting partial impairment of CSF absorption [50]. The authors also demonstrated the value of the test in distinguishing arrested from progressive hydrocephalus in an infant with ventriculomegaly and developmental delay, highlighting its potential role in the clinical evaluation of complex disorders of CSF circulation [50]. Among 14 patients with dementia, most individuals with clinically or pathologically confirmed AD had normal infusion-test results [50]. However, one patient with AD exhibited both abnormal CSF dynamics and radiological features of communicating hydrocephalus, an observation that anticipated the current recognition that AD and iNPH frequently coexist and that concomitant neurodegenerative pathology may influence the response to shunt surgery [50].

### 4.4. From Absorptive Capacity to Resistance to CSF Outflow (R_out_)

Although Katzman and Hussey pioneered the lumbar infusion test (LIT), they did not explicitly formulate the concept of resistance to CSF outflow (R_out_). Rather, they viewed the test as an assessment of CSF absorptive reserve, emphasizing the capability of the absorptive pathways to accommodate fluid loads well above physiological CSF production [18,50]. A significant methodological advance followed in 1971, when Nelson and Goodman introduced strain-gauge pressure transducers and continuous ICP recording, improving measurement accuracy and simplifying the procedure [51]. They further demonstrated that an infusion rate of 1.5 mL/min provided optimal discrimination between normal and impaired CSF absorption [51].

Like Katzman and Hussey, Nelson and Goodman did not use resistance to CSF outflow (*R_out_*) to distinguish between patient groups. Instead, they evaluated the ICP response to infusion in patients with suspected defects in CSF absorption, pseudotumor cerebri, and healthy volunteers. Patients were classified according to the rate of CSF pressure increase during infusion. Healthy individuals consistently exhibited pressure increases of less than 20 mmH_2_O/min at an infusion rate of 1.5 mL/min, whereas patients with impaired CSF absorption showed substantially greater pressure elevations [51]. These findings suggested that abnormalities of CSF absorption could be identified from the ICP response to infusion even before the formal introduction of *R_out_* as a quantitative hydrodynamic parameter. With the development of Marmarou’s pressure-flow model, the concept of CSF absorptive capacity was reformulated as a measurable hydraulic parameter, namely resistance to CSF outflow (*R_out_*), which is equal to the reciprocal of conductance to CSF outflow (*C_out_*).

### 4.5. From Davson’s Equation to Marmarou’s Model

Davson’s work established that CSF absorption is a pressure-dependent process driven by the pressure gradient between ICP and the venous system, analogous to the flow of fluid through a resistive pathway as described by Poiseuille’s law [32]. In the literature, Equation (1)—the principal mathematical model describing steady-state ICP—has traditionally been referred to as Davson’s equation [52]. However, our review of the original writings of Davson and Marmarou suggests that the equation, in the form now used in infusion studies, may have been first explicitly formulated by Marmarou in his 1973 PhD thesis [53,54]. While its physiological basis clearly originates from Davson’s work, the mathematical derivation and subsequent application to CSF hydrodynamics (Equation (1)) appear to have been developed by Marmarou.

*ICP* = (*If* × *R_out_*) + *P_ss_* (1)

where *ICP* = intracranial pressure, *If* = CSF formation rate (mL/min), *R_out_* = resistance to CSF outflow (mmHg·min/mL) and *Pss* = superior sagittal sinus pressure (mmHg). A key assumption underlying this equation is that pressure within the superior sagittal sinus (P_ss_) remains relatively constant. As Davson argued, *“The fact that the dural sinuses are enclosed within the tough dura suggests that they are unlikely to be seriously affected by changes in intracranial pressure.”* [32].

Although Davson recognized that CSF is continuously produced, endogenous CSF formation was not treated as an explicit variable in the equations he used to estimate resistance. Rather, resistance was inferred from the relationship between ICP and CSF outflow during infusion, under the assumption that CSF production remained essentially constant throughout the measurement period [32]. While the physiological concepts underpinning Equation (1) originate largely from Davson’s work, the notation and mathematical formulation employed in contemporary CSF hydrodynamics are generally attributed to Marmarou. In this formulation, *I_f_* represents the rate of CSF formation, *R_out_* the resistance to CSF outflow, and *P_ss_* the superior sagittal sinus pressure [53,54]. Accordingly, increases in *R_out_* are predicted to elevate ICP, providing the theoretical basis for infusion studies designed to quantify CSF absorptive capacity [53,54].

In most clinical situations, I_f_ is assumed to remain relatively constant, and it is widely accepted that clinically significant increases in CSF production are uncommon, occurring primarily in rare conditions such as choroid plexus papilloma. Consequently, abnormalities in CSF dynamics are generally attributed to alterations in CSF absorption rather than to changes in CSF production. In Marmarou’s equation (Equation (1)), *P_ss_* denotes the pressure within the superior sagittal sinus and represents the downstream pressure opposing CSF absorption through the arachnoid villi and granulations. As defined by this model, ICP is determined not only by the rate of CSF formation (*I_f_*) and the *R*_out_ but also by the pressure within the venous sinuses, represented by the superior sagittal sinus pressure (*P_ss_*), as originally proposed by Davson [32]. The clinical appeal of Equation (1) lies in its simplicity. By assuming that *I*_f_ and *P*_ss_ remain constant across individuals and throughout the infusion study, ICP becomes largely a function of *R*_out_, effectively reducing a complex physiological system to a single measurable parameter. This simplification made infusion testing practical for routine clinical use and established *R*_out_ as the principal hydrodynamic biomarker derived from the LIT.

Building on Davson’s seminal work [32], Marmarou et al. pioneered the application of mathematical and simulation-based approaches to the study of CSF dynamics, integrating experimental physiological observations with quantitative modeling of the intracranial system [53,54]. They developed a conceptual representation of CSF formation and absorption, translated it into mathematical equations, employed electrical circuit analogies for analytical purposes, and implemented these models computationally to simulate CSF dynamics, explicitly incorporating intracranial compliance as a key parameter [53,54]. In his seminal PhD work published in 1973, Anthony Marmarou introduced the *bolus injection test* and performed a series of constant-rate infusion experiments in cats. His studies demonstrated that the rate of ICP increase is determined by both intracranial compliance and *R*_out_ whereas the final steady-state pressure depends solely on the absorptive properties of the CSF system. Building on the pioneering studies of Pappenheimer et al. [41] and Davson’s seminal work on CSF physiology [32], Marmarou developed a mathematical model of CSF dynamics that incorporated intracranial compliance. The resistance to CSF absorption, later termed the resistance to CSF outflow, and denoted as *R*_out_ in Equation (1), can be derived and expressed as shown in Equation (2).

(2) $$R_{\mathrm{out}}=\frac{\mathrm{Pp}-P0}{I}$$

where *P_p_* is the plateau pressure reached during infusion, *P_o_* is the opening pressure before infusion, and *I* is the infusion rate expressed in mL/min. Thus, *R*_out_ represents the pressure increase required to sustain a unit increase in CSF flow [53]. As Marmarou stated, “According to the system equations, for constant pressure in the dural sinus, the change in steady-state level divided by the change in infusion represents the effective resistance to absorption.” [53].

### 4.6. Defining Normal Rout: Reference Ranges and Clinical Implications

Resistance to CSF outflow (*R_out_*) has traditionally been regarded as both a diagnostic and prognostic biomarker in iNPH. It has been used to identify patients most likely to benefit from CSF shunting and, in some studies, to help distinguish iNPH from cerebral atrophy and other causes of *ex vacuo* ventriculomegaly. However, more than five decades after the introduction of the lumbar infusion test (LIT), considerable debate remains regarding both the normal reference range of *R_out_* and the threshold most appropriate for predicting postoperative improvement in patients with iNPH. This lack of consensus reflects not only methodological heterogeneity among studies but also the limited availability of robust normative data, particularly in elderly individuals, in whom age-related increases in *R_out_* may substantially overlap with values reported in patients with iNPH.

In a study of eight healthy volunteers, Albeck et al. (1991) established the first normative values for CSF hydrodynamics using a lumbar constant-pressure technique [55]. They reported a mean ICP of 11 ± 2 mmHg and a mean CSF outflow conductance (*C*_out_) of 0.11 mL/min/mmHg, equivalent to an *R*_out_ of approximately 9.1 mmHg·min/mL [55]. These findings further supported a linear relationship between ICP and CSF absorption and provided independent validation of earlier observations derived from clinical populations [55]. In a second study (1998), Albeck et al. used the LIT in 52 individuals aged 20–88 years without known CSF disorders who were undergoing elective orthopedic or abdominal surgery under spinal anesthesia thereby providing a unique opportunity to establish age-related normative data [56]. This influential study confirmed that the mean *R*_out_ in participants younger than 30 years was 10.8 mmHg·min/mL, consistent with earlier findings from the same group, and demonstrated that while mean ICP remained largely unchanged with age, *R*_out_ increased linearly according to the equation *Rout* = 9.88 + 0.075 × age [56]. These findings indicate that *R*_out_ increases progressively with age, reaching values approximately 5 mmHg·min/mL higher in octogenarians than in young adults. The authors proposed that this age-related increase in *R*_out_ may be attributable to degenerative changes or subclinical leptomeningeal obstruction and warned that the application of a fixed diagnostic threshold—such as 12 mmHg·min/mL—may misclassify healthy elderly individuals as having impaired CSF absorption, thereby increasing the risk of unnecessary shunting [56]. These observations were subsequently extended by Malm et al. (2011), who evaluated CSF hydrodynamics in 40 healthy individuals aged 60–82 years, all of whom underwent Magnetic resonance imaging (MRI) within 24 h of testing [57]. They found a median ICP of 11.6 mmHg (reference interval, 7.8-14.3 mmHg), comparable to values reported in younger populations. The mean *R_out_* was 11.1 mmHg·min/mL; however, the upper reference limit reached 17.4 mmHg·min/mL (90th percentile), substantially exceeding previous estimates for this age group [57]. Neither ventricular size, cerebral atrophy, nor white matter lesion burden correlated with ICP or *R_out_*. Notably, 10% of these healthy elderly individuals exhibited R_out_ values meeting a commonly used threshold for shunt surgery (≥18 mmHg·min/mL) [57]. Taken together, these findings indicate that elevated *R_out_* is not specific to iNPH and should be interpreted as a marker of altered CSF dynamics rather than a definitive indicator of disease. More broadly, they highlight the importance of interpreting *R_out_* within the context of age, clinical presentation, neuroimaging findings, and complementary investigations, rather than relying solely on fixed diagnostic thresholds.

## 5. Alternative Infusion-Based Methods for Assessing CSF Dynamics

The pioneering work of Hakim et al. sparked considerable interest in identifying patients with iNPH who might benefit from shunt surgery. However, the diagnostic tools available during the latter half of the twentieth century were limited, making patient selection a major clinical challenge. In particular, radioisotope cisternography proved insufficiently specific, as ventricular reflux, a common finding in communicating hydrocephalus, could also be observed in patients with cerebral atrophy. This limitation stimulated the search for more objective methods of assessing CSF hydrodynamics and contributed to the widespread adoption of the LIT as a clinical tool for evaluating CSF absorptive function. Around the same time that Katzman and Hussey introduced their infusion test, several alternative techniques were developed to assess CSF dynamics in iNPH, including ventriculocisternal (or ventriculolumbar) perfusion, Ekstedt’s constant-pressure infusion method, and Marmarou’s bolus test. Despite the physiological insights generated by these techniques, the LIT became the dominant method for assessing CSF absorptive capacity in iNPH and achieved widespread clinical acceptance. Alternative approaches remained largely confined to specialized centers and were not widely adopted in routine practice or incorporated into contemporary diagnostic guidelines. Nevertheless, they warrant brief review because of their historical importance and contribution to the development of modern concepts of CSF hydrodynamics.

### 5.1. Assessment of CSF Compliance and Absorption: The Sokolowski Technique

Working independently of Marmarou, Sokolowski developed a repeated-bolus injection method that foreshadowed several aspects of contemporary CSF hydrodynamic testing [58]. In a 1974 report, he described a bedside technique intended to provide quantitative estimates of “*the rate of CSF formation, rate of absorption, resistance to absorption, and compliance factor*” [58]. Unlike Marmarou, Sokolowski employed a repeated-bolus technique and analyzed successive pressure–time segments to derive estimates of *R*_out_ and intracranial compliance (*C*), a parameter describing the capacity of the intracranial compartment to accommodate volume changes with minimal alterations in ICP. Although innovative and ahead of its time, Sokolowski’s method was eventually replaced by more standardized techniques, including the LIT and Marmarou’s bolus injection test, which offered greater reproducibility and a more comprehensive characterization of CSF hydrodynamics.

### 5.2. Ekstedt’s Constant Pressure Infusion Method

Introduced by Ekstedt in 1977, the constant-pressure infusion technique offered an alternative to the Katzman-Hussey test. Instead of infusing fluid at a fixed rate, CSF pressure was maintained at a predetermined level while the flow required to sustain that pressure was measured [59]. Using artificial CSF and a two-needle system for independent pressure monitoring, the method enabled estimation of C_out_, CSF formation rate (*I_f_*), and superior sagittal sinus pressure (*P_ss_*), while providing further evidence of a linear pressure-flow relationship within the CSF system [59].

The principal limitation of Ekstedt’s method was its technical complexity. The requirement for a servo-controlled infusion system, continuous flow monitoring, and a two-needle setup for independent pressure measurement made it substantially more demanding than the LIT, limiting its use largely to specialized research centers. Despite this complexity, the technique showed excellent agreement with simpler infusion methods. In a comparative study of 16 patients, Børgesen et al. found that values obtained with the constant-pressure method were only slightly lower than those derived from a computerized LIT, with differences remaining within the 95% confidence limits [60]. These findings suggested that the simpler LIT could provide clinically equivalent information while being considerably easier to perform.

### 5.3. Marmarou’s Bolus Test

**Figure 3:**
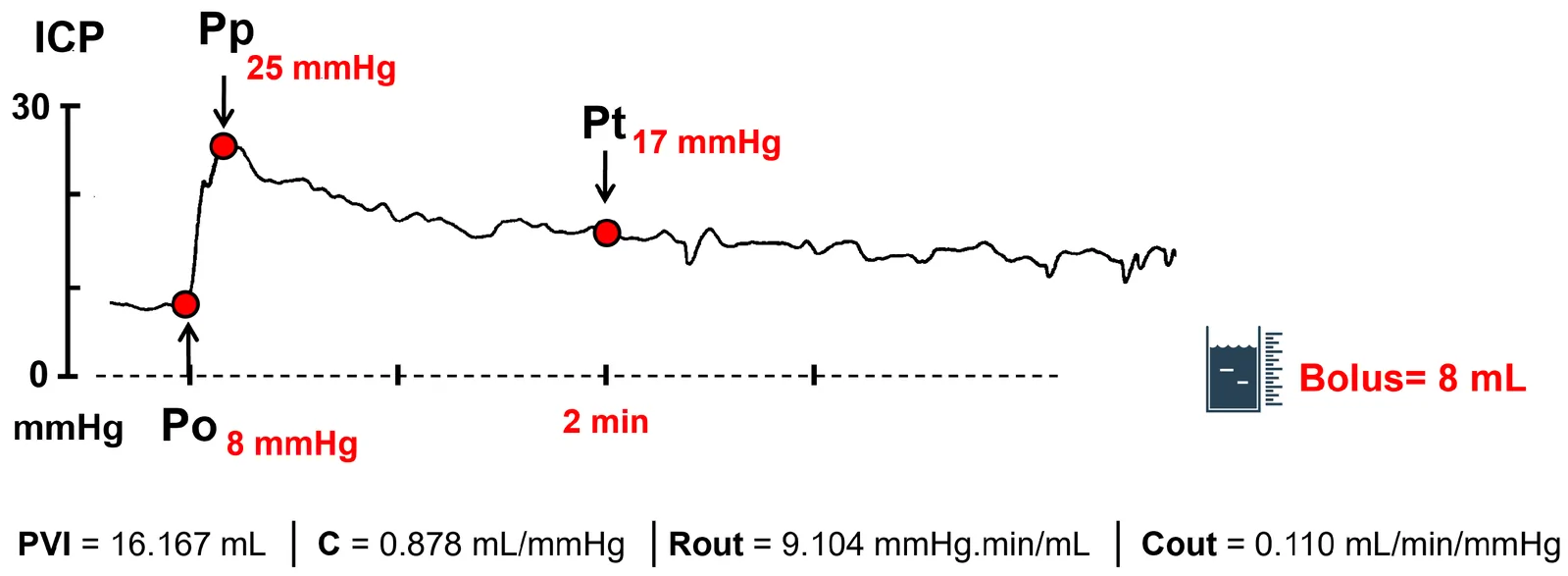
Marmarou’s bolus test. A total volume of 8 mL of normal saline was injected at an approximate rate of 1 mL/s. The graph illustrates the baseline intracranial pressure (P_0_), the peak pressure immediately after bolus injection (*Pp*), and the pressure measured 2 min later (Pt). This test provides a rapid assessment of intracranial compliance (*C*) and resistance to cerebrospinal fluid outflow (*R*out). In this example, the calculated values were a pressure–volume index (PVI) of 16.2 mL, a compliance of 0.9 mL/mmHg, an *R*out of 9.1 mmHg·min/mL, and a CSF outflow conductance (*C*out) of 0.110 mL/min/mmHg.

Particular mention should be made of Marmarou’s bolus test, which we have employed for many years alongside the LIT in the study of iNPH. In this method, the peak ICP response following a rapid volume injection is used to assess intracranial compliance, while the subsequent pressure decay (transient phase) is analyzed to characterize absorptive capacity [53,54,61]. In brief, the Marmarou bolus test is a quantitative method for assessing CSF hydrodynamics through the measurement of intracranial compliance and *R*_out_ following the rapid injection of a known fluid volume (usually 8 mL in adults) [61] (Figure 3). Building on his nonlinear mathematical model of the CSF system, Marmarou demonstrated that the relationship between ICP and intracranial volume is exponential, indicating that compliance decreases as pressure increases. To simplify this relationship, he introduced the Pressure–Volume Index (PVI), defined as the volume required to increase resting ICP tenfold on a semilogarithmic pressure–volume curve [54].

During the test, the injected bolus volume, baseline ICP, and peak ICP are used to calculate the PVI, providing a quantitative measure of intracranial compliance (Figure 3). The subsequent pressure decay, as the added fluid is absorbed and ICP returns toward equilibrium, is then analyzed to estimate *R*_out_ [54]. For reasons that remain incompletely understood, *R*_out_ values derived from Marmarou’s bolus technique are consistently lower than those obtained with the LIT [62]. This discrepancy likely reflects differences in the underlying mathematical models, the influence of intracranial compliance, and the distinct physiological conditions under which the two techniques assess CSF dynamics.

In a study of 30 adults without intracranial pathology, Sahuquillo et al. established normative values for CSF hydrodynamic parameters using Marmarou’s bolus test [62]. Mean baseline ICP was 8.3 mmHg, mean pressure-volume index (PVI) was 20.3 mL, and mean *R*_out_ was 3.4 mmHg·min/mL. Based on these findings, the authors proposed an *R*_out_ threshold > 5 mmHg·min/mL as indicative of impaired CSF absorption.

## 6. Current Controversies of the LIT in iNPH

The theoretical basis of the LIT rests on the assumption that impaired CSF absorption is a fundamental physiological abnormality in iNPH. This disturbance can be quantified by *R*_out_ or its reciprocal, *C*_out_, which serve as surrogate measures of CSF absorptive capacity. Although these parameters provide objective and reproducible indicators of CSF hydrodynamics, their interpretation remains controversial. Five decades after the introduction of the LIT, uncertainty persists regarding the physiological significance of elevated *R*_out_ values, the optimal threshold for predicting shunt responsiveness, and the extent to which infusion-test results should influence clinical decision-making.

**Figure 4:**
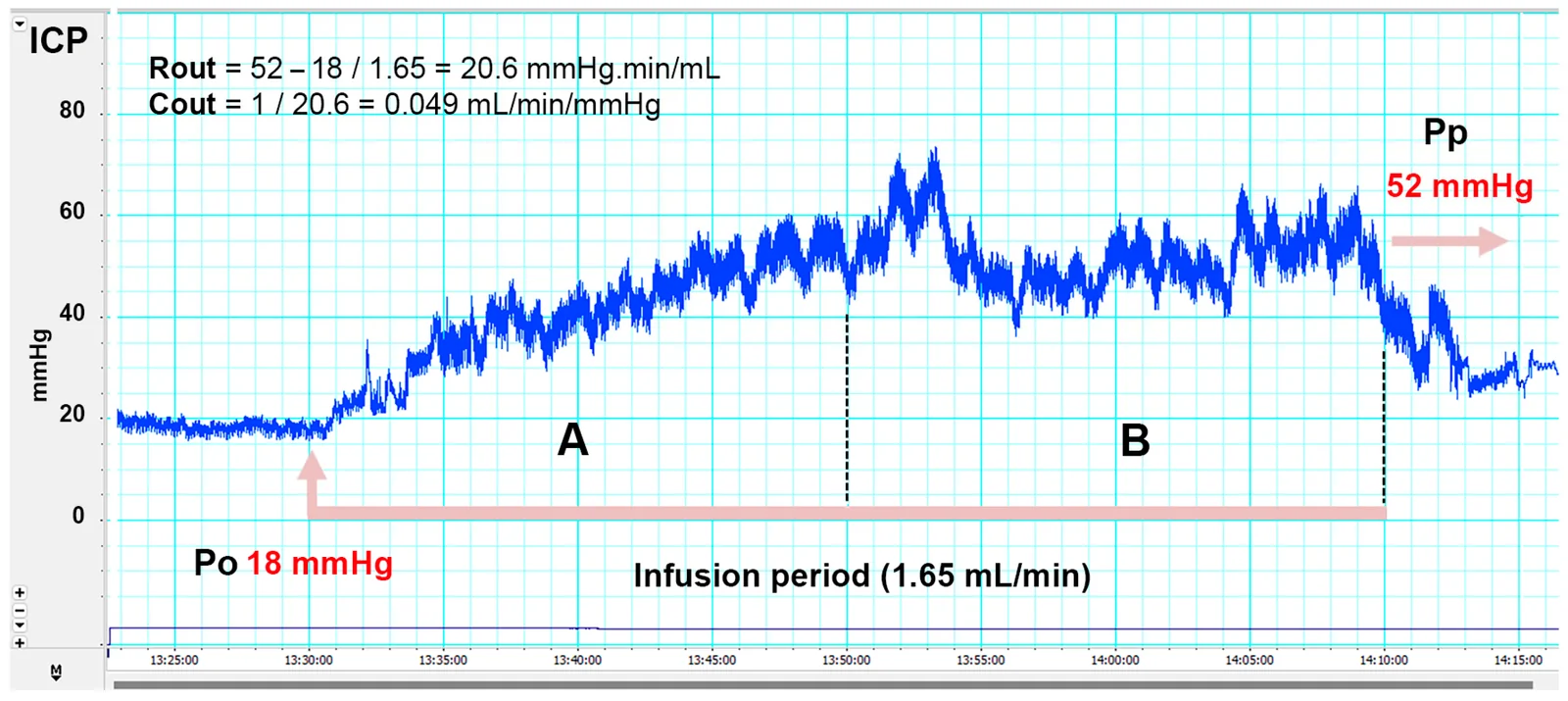
Katzman infusion test performed using a continuous infusion of Ringer’s lactate at 1.65 mL/min. Baseline intracranial pressure (ICP) was 18 mmHg (*Po*), and the mean plateau pressure was 52 mmHg (*Pp*). The total infusion period extended from 13:30 to 14:10. Segment A shows the phase of progressive increase in ICP, whereas segment B corresponds to the plateau phase. The resistance to cerebrospinal fluid outflow (*R*_out_) was 20.6 mmHg·min/mL, and the corresponding conductance to outflow (Cout = 1/*R*_out_) was 0.049 mL/min/mmHg.

During the LIT, fluid infusion increases ICP and thereby augments the pressure gradient that drives CSF absorption through bulk flow across the absorptive pathways. A new steady state is reached when the combined inflow from endogenous CSF production and external infusion is balanced by CSF outflow, resulting in a plateau pressure (Figure 4). Although the intracranial compartment is traditionally regarded as non-distensible and its contents largely incompressible under the Monro–Kellie doctrine [63,64], modest increases in intracranial volume can be accommodated through displacement of CSF into the spinal compartment, expansion of the spinal dural sac, and redistribution of venous blood. Once this steady state has been achieved, Equation (2) can be used to calculate *R*_out_ or its reciprocal, *C*_out_ (=1/*R*_out_) [60,65]. Although infusion testing allows estimation of several physiological parameters, *R*_out_ remains the principal biomarker of clinical interest and forms the basis of the conventional use of the LIT in patients with suspected iNPH [65]. In one of the earliest studies investigating the role of infusion testing in suspected NPH, Wolinsky et al. (1973) evaluated the diagnostic and prognostic utility of pneumoencephalography, radioisotope cisternography, and the lumbar infusion test (LIT) in 22 patients presenting with dementia and ventriculomegaly [66]. Using the Katzman–Hussey criterion, they considered a plateau pressure above 300 mmH_2_O (approximately 22 mmHg) indicative of impaired CSF absorption and supportive of a diagnosis of NPH [66]. However, the LIT showed limited predictive value: several patients with normal test results improved after VA shunting, whereas some with abnormal results did not benefit from surgery. Consequently, the authors regarded the test as “not useful” for clinical decision-making in their series [66]. In 1980, Vastola retrospectively analyzed a heterogeneous cohort of patients with suspected iNPH and proposed a lower normal limit for *C*_out_ of 0.007 mL/min/mmH_2_O, corresponding to an *R*_out_ of approximately 10.5 mmHg·min/mL in contemporary units [67]. This represented one of the earliest attempts to define a quantitative hydrodynamic criterion for selecting patients for shunt surgery.

In a subsequent study, Børgesen et al. (1992) highlighted important limitations of the LIT, noting that “*a plain lumbar infusion at constant rate (so-called Katzman test) is easy to perform but the interpretation of the results is difficult and the obtained values often unreliable*” [60]. Despite these concerns, available evidence suggests that the LIT is highly reproducible. Czepko and Cieślicki evaluated the reproducibility of *R*_out_ in 27 patients with normal-pressure hydrocephalus by repeating the test after a mean interval of 5.6 months [68]. The close agreement between repeated *R*_out_ measurements obtained at an infusion rate of 2.0 mL/min in the same patients demonstrated excellent test–retest reliability, supporting the stability of this parameter over time. Despite this reproducibility, the search for a clinically meaningful *R*_out_ threshold remains unresolved. A major objective has been to identify a threshold that reliably distinguishes iNPH from ventriculomegaly associated with cerebral atrophy (hydrocephalus *ex vacuo*) while accurately predicting postoperative improvement following shunt surgery.

### Integration of the LIT into Clinical Practice Guidelines for iNPH

During the 1990s, the emergence of Evidence-Based Medicine (EBM) profoundly influenced clinical decision-making and stimulated the development of evidence-based clinical practice guidelines (CPGs) [69]. By integrating the best available evidence with clinical expertise, CPGs sought to standardize care and promote more objective and reproducible approaches to diagnosis and treatment [70]. In iNPH, the increasing use of ancillary investigations, including the LIT, CSF tap test, ICP monitoring, and external lumbar drainage, prompted efforts to clarify their diagnostic and prognostic value and establish evidence-based criteria for patient selection.

The first international CPGs for iNPH published in English were developed by Marmarou et al. in 2005 and became known as the International, or US, Guidelines [20]. Together with the first Japanese guidelines published in 2004 [71], they marked the beginning of evidence-based approaches to iNPH management. At present, three major guideline documents inform clinical practice: (1) the International (US) Guidelines (2005), (2) the Japanese Guidelines, currently in their third English-language edition (2021), and (3) the American Academy of Neurology (AAN) guidelines published in 2015 [4,19,24,71]. Although the role assigned to the LIT varies among these documents, all recognize it as an important ancillary investigation in patients with suspected iNPH.

In 2013, Wikkelsø et al. reported the European Multicentre Study on iNPH (Eu-iNPH), one of the largest prospective investigations of the prognostic value of *R*_out_ in suspected iNPH [21]. Conducted across 13 centers in nine European countries, the study demonstrated that neither elevated *R*_out_ nor a positive CSF tap test reliably predicted the magnitude of clinical improvement 12 months after surgery [21]. Although both tests were useful for identifying patients likely to benefit from CSF diversion, a negative result did not reliably exclude a favorable postoperative outcome and therefore could not be used as a basis for withholding treatment [21]. Taken together, the major studies and guideline documents reviewed here suggest that the LIT provides useful physiological and prognostic information but should generally be interpreted as an adjunctive investigation rather than a stand-alone determinant of shunt eligibility. Although elevated *R*_out_ values may increase the probability of postoperative improvement, negative test results do not reliably exclude a favorable response to shunt surgery.

Interpreting the significance of these findings requires an understanding of the metrics used to evaluate diagnostic and prognostic test performance. Before examining the available evidence on *R*_out_ thresholds in greater detail, it is therefore useful to review the principles underlying diagnostic accuracy, prognostic performance, and their interpretation in clinical practice.

## 7. Assessing Diagnostic and Prognostic Test Performance

As emphasized by Sackett in his classic discussion of diagnostic testing, ancillary investigations in patients with suspected iNPH serve purposes that extend beyond simply supporting or refuting the diagnosis [69]. They may contribute to disease staging and prognostic stratification, particularly with respect to responsiveness to CSF shunting. Ultimately, however, their clinical utility depends on how accurately they identify patients likely to benefit from surgery and distinguish them from those unlikely to improve.

As Deeks et al. observed, the value of a diagnostic test lies not solely in its accuracy, but in its ability to improve patient outcomes. As they noted, “*more accurate tests will improve patient outcomes if the reductions in false positive or false negative results lead to more people receiving appropriate diagnoses and appropriate treatment*” [27,72,73]. The diagnostic performance of a test is conventionally evaluated by comparing its results with a reference method capable of establishing the true disease status (the “reference standard” or “gold standard”) [72,74,75,76]. Diagnostic accuracy is commonly summarized using a 2 × 2 contingency table (Table 1), from which sensitivity (S), specificity (Sp), positive predictive value (PPV), and negative predictive value (NPV) can be calculated [25,73]. Although these metrics are widely used, their interpretation is often misunderstood and inconsistently reported in the clinical literature [77]. To improve methodological quality and transparency in diagnostic studies, the STARD 2015 (Standards for Reporting Diagnostic Accuracy Studies) statement was developed [77]. The identification, appraisal, and reporting of diagnostic accuracy data in this review were guided by these recommendations.

Applied to *R*_out_ in iNPH, S and Sp quantify the ability of a given threshold to discriminate between responders and non-responders according to the surrogate reference standard of postoperative improvement following shunt surgery [25]. In the hypothetical example shown in Table 1, an *R*_out_ threshold of 12 mmHg·min/mL yielded a sensitivity of 92.8% and a specificity of 69.7%. Thus, most responders were correctly classified as test-positive, whereas approximately 70% of non-responders were correctly classified as test-negative. PPV and NPV address a different question by estimating the probability of treatment response following a positive or negative test result, respectively [25,26,72,73].

In the example shown in Table 1, the PPV is 90% and the NPV is 76.7%. While these measures are intuitive and clinically relevant, they are strongly influenced by disease prevalence and therefore vary across patient populations and clinical settings [25,26,72,73]. As Guyatt et al. emphasized, different clinical settings inevitably evaluate patients with different pre-test probabilities of disease [78]. In iNPH, these probabilities vary considerably across the referral pathway, from primary care patients with isolated gait disturbance, urinary symptoms, or mild cognitive impairment to highly selected patients referred to specialist dementia clinics and tertiary neurosurgical centers for LIT or ICP monitoring. Consequently, the predictive significance of a positive or negative LIT result depends not only on the intrinsic performance of the test but also on the prevalence of shunt-responsive iNPH in the population under consideration. This dependence on prevalence limits the generalizability of predictive values and has motivated the adoption of Bayesian approaches, which explicitly incorporate pre-test probability into the interpretation of test results. Addressing this limitation requires methods that combine pre-test probabilities with test-specific likelihood ratios (LRs) to estimate how LIT findings modify the probability of postoperative shunt responsiveness [72,73].

## 8. Bayesian Interpretation of Diagnostic Tests

LRs provide the quantitative link between pre-test and post-test probability within a Bayesian framework. Derived from S and Sp, they quantify how much a positive or negative test result changes the probability that a patient has the target condition [26,73,79]. Unlike predictive values, LRs are largely independent of disease prevalence and can therefore be applied across different clinical settings. By combining an estimated pre-test probability with the appropriate LR, clinicians can calculate the corresponding post-test probability, making LRs particularly useful for individualized diagnostic decision-making [26,27,80]. The positive likelihood ratio (LR+) is defined as the probability of a positive test result in individuals with the target condition divided by the probability of a positive test result in those without the condition and is calculated as:

$$\left(\mathrm{LR}+\right)=\frac{S}{1-\mathrm{Sp}}$$

Conversely, the negative likelihood ratio (LR−) is defined as the probability of a negative test result in individuals with the target condition divided by the probability of a negative test result in those without the condition [26,27,72,73,80] and is calculated as:

$$\left(\mathrm{LR}-\right)=\frac{1-S}{\mathrm{Sp}}$$

An LR greater than 1 increases the probability of the target condition, whereas an LR less than 1 decreases it. The further an LR departs from 1, the greater its effect on post-test probability and, consequently, its clinical utility [26,79,80]. Conventionally, LR+ values greater than 10 and LR− values less than 0.1 provide strong evidence for ruling in and ruling out a condition, respectively, whereas LR+ values between 2 and 5 and LR− values between 0.5 and 0.2 produce only small to moderate changes in probability and rarely alter clinical decisions when considered in isolation [26,27,73]. For example, in a patient with a pre-test probability of shunt responsiveness of 50%, an LR+ of approximately 3 would increase the post-test probability to approximately 75%. This illustrates that elevated *R*_out_ values may meaningfully increase the probability of treatment responsiveness but do not provide definitive evidence of a favorable postoperative outcome.

Within the Bayesian framework, post-test odds are obtained by multiplying pre-test odds by the likelihood ratio corresponding to the observed test result. This approach enables clinicians to estimate how a given *R*_out_ threshold modifies the probability of shunt responsiveness in an individual patient [80]. Because LRs are largely independent of disease prevalence, they can be applied across populations with different baseline risks, provided that sensitivity and specificity remain stable. Consequently, the clinical usefulness of a particular *R*_out_ threshold depends not only on its sensitivity and specificity but also on the likelihood ratios it generates and the resulting change from pre-test to post-test probability. Readers seeking a more comprehensive discussion of these concepts are referred to the authoritative review by Deeks and Altman [27].

## 9. The Persistent Search for an Optimal *R_out_* Threshold

Despite decades of investigation, no consensus has been reached regarding the optimal *R*_out_ threshold for predicting improvement following CSF shunting. This lack of agreement reflects the inherent trade-off between sensitivity and specificity associated with different cutoff values and has generated considerable uncertainty regarding the clinical utility of the LIT and the extent to which *R*_out_ can serve as a reliable predictor of shunt responsiveness. Reported thresholds range from approximately 10 to 18 mmHg·min/mL, reflecting substantial heterogeneity in patient selection, infusion methodology, reference standards, and outcome definitions across studies [81,82].

The Dutch Normal-Pressure Hydrocephalus Study (1997) was a landmark prospective multicenter trial involving 101 patients, all of whom underwent shunt surgery regardless of their measured *R*_out_ [81]. This study enabled a robust evaluation of the prognostic value of LIT across the full spectrum of *R_out_* values. Although *R*_out_ did not correlate with the magnitude of postoperative improvement, elevated values remained useful for identifying patients more likely to benefit from shunt surgery. An *R*_out_ threshold of 18 mmHg·min/mL yielded a PPV of 92% and an LR+ of 3.5, whereas lower thresholds were associated with poor NPVs because many patients with lower *R*_out_ values nevertheless improved after shunt surgery. Approximately 60% of patients experienced clinically worthwhile improvement at one year. These findings suggest that elevated *R*_out_ values may support patient selection, but that *R*_out_ alone cannot be used to exclude potentially shunt-responsive patients [81].

The first meta-analysis specifically evaluating the prognostic value of *R*_out_ in iNPH was conducted by Kim et al. in 2015 [83]. To address the long-standing uncertainty surrounding the optimal *R_out_* threshold for predicting clinical improvement after shunt surgery, the authors analyzed data from nine studies published between 1986 and 2013 [83]. Both *R_out_* thresholds of 12 and 18 mmHg·min/mL were associated with high positive predictive values for postoperative improvement. Among the thresholds evaluated, 12 mmHg·min/mL demonstrated the best overall diagnostic performance, with an accuracy of 72.9% and a sensitivity of 80.3%, although Sp remained only moderate (46.8%) [83]. These findings suggested that lower *R_out_* thresholds are more effective for identifying patients likely to benefit from shunt surgery but do so at the expense of an increased false-positive rate, thereby limiting their overall discriminatory performance [83].

A second meta-analysis, published by van Bilsen et al. in 2025, adopted a Bayesian perspective to examine whether the LIT could function as a reliable rule-out test by identifying patients with iNPH who were unlikely to benefit from shunt surgery [82]. The authors pooled data from 10 studies and evaluated the negative predictive values associated with the most commonly used *R*_out_ thresholds [82]. However, differences in study selection, inclusion criteria, and analytical methodology between the two meta-analyses precluded direct comparison or formal pooling of their results [82,83]. Although both meta-analyses reported pooled estimates of sensitivity and specificity for the investigated *R*_out_ thresholds, neither presented the corresponding likelihood ratios. Therefore, we extracted the pooled sensitivity and specificity estimates from both meta-analyses and calculated the corresponding likelihood ratios for each proposed *R*_out_ threshold.

The resulting values, summarized in Table 2, facilitate a comparative Bayesian interpretation of the reported prognostic performance of the different *R*_out_ thresholds for predicting shunt responsiveness. Because the thresholds summarized in Table 2 were derived from different meta-analyses and underlying study populations, the reported values should not be interpreted as direct threshold comparisons within a single cohort. To our knowledge, this is the first study to derive the likelihood ratios associated with the *R*_out_ thresholds evaluated in published meta-analyses and to compare their implications within a unified Bayesian framework, thereby providing a unified interpretation of their reported prognostic performance and their potential contribution to clinical decision-making.

As shown in Table 2, none of the *R*_out_ thresholds evaluated in either meta-analysis generated likelihood ratios associated with large shifts in post-test probability. LR+ ranged from only 1.15 to 1.72, while LR- ranged from 0.41 to 0.81. Consequently, although elevated *R*_out_ values may increase the probability of shunt responsiveness, the magnitude of this effect appears modest when *R*_out_ is considered in isolation. According to conventional interpretation, these values produce only small changes in post-test probability and are insufficient to confidently rule in or rule out shunt responsiveness [26,27,73]. Although lower *R*_out_ thresholds generally achieved higher S and higher thresholds improved Sp, these trade-offs did not translate into clinically meaningful likelihood ratios. Consequently, the available evidence does not support the use of any single *R*_out_ threshold as a stand-alone predictor of postoperative improvement. Accordingly, the available evidence indicates that *R*_out_ should not be used as a stand-alone predictor but should always be interpreted in conjunction with the patient’s clinical presentation, neuroimaging findings, and other diagnostic investigations.

## 10. The Reference Standard Dilemma in iNPH

The consistently low LRs observed across all proposed *R*_out_ thresholds raise an important question: why has a physiological parameter with excellent test–retest reliability failed to emerge as a robust predictor of postoperative outcome? A major contributor to this apparent discrepancy is the reference standard against which *R*_out_ has traditionally been evaluated. Unlike many areas of medicine, iNPH lacks a universally accepted diagnostic gold standard. Consequently, most studies assessing the diagnostic and prognostic performance of ancillary investigations, including the LIT, CSF tap test, external lumbar drainage, and ICP monitoring, have adopted postoperative clinical improvement following shunt surgery as a surrogate reference standard. As discussed previously and emphasized by others [6,7], this approach introduces important methodological limitations because postoperative outcome is influenced by numerous factors beyond CSF hydrodynamics, including patient selection, concomitant neurodegenerative disorders, surgical technique, shunt type and valve settings, shunt-related complications, outcome definitions, and duration of follow-up. Consequently, postoperative improvement represents an imperfect reference standard not only for evaluating the prognostic value of *R*_out_ but also for confirming the diagnosis of iNPH, a practice that has nevertheless been endorsed by the latest edition (2021) of the Japanese Clinical Practice Guidelines despite its recognized limitations [4].

A fundamental challenge in iNPH is that no universally accepted reference standard exists. Neither clinical features, neuroimaging findings, physiological tests, nor neuropathological observations have been universally accepted as a definitive diagnostic gold standard. Consequently, ancillary investigations such as the LIT are evaluated primarily according to their ability to predict shunt responsiveness rather than to establish the diagnosis itself. Although this approach in iNPH is imperfect and has attracted criticism, it is consistent with the principles of Evidence-Based Medicine, which hold that the ultimate value of a diagnostic test lies in its ability to improve patient outcomes rather than in its diagnostic accuracy alone [69,78]. Nevertheless, this approach creates a methodological paradox: the diagnosis is effectively validated by the patient’s response to a treatment that was undertaken because the diagnosis was presumed to be correct in the first place [6,7]. Thus, postoperative improvement functions simultaneously as both the outcome being predicted and the surrogate reference standard against which predictive tests are evaluated. This dual role introduces an inherent risk of circular reasoning and complicates interpretation of the reported diagnostic performance of *R*_out_. Because of this reliance on an imperfect reference standard, the calculated sensitivity, specificity, and likelihood ratios are influenced not only by the intrinsic performance of the LIT but also by how postoperative outcome is defined, assessed, and measured.

### The Challenge of Defining Non-Response

It is important to distinguish between the diagnostic role of ancillary investigations in supporting the diagnosis of iNPH and their prognostic role in predicting responsiveness to CSF shunting. In practice, however, these two objectives are closely intertwined because postoperative improvement has frequently been used as a surrogate reference standard for evaluating both. A further consequence of relying on postoperative improvement as a surrogate reference standard is the absence of standardized outcome definitions, particularly the considerable variability in the criteria used to define shunt responsiveness across studies.

Studies that define shunt responsiveness as any subjectively perceived clinical improvement typically report higher response rates and, consequently, higher positive predictive values for a given threshold than studies requiring objective, quantitative outcomes assessed at predefined follow-up intervals by independent evaluators. Moreover, the scales used to define postoperative improvement vary substantially. The NPH scale, the modified Rankin Scale used in the Dutch NPH Study, and the three-category clinical improvement scale used in the Cambridge cohorts measure different constructs and apply different thresholds for defining response. This lack of standardization complicates comparisons across studies and is likely a major contributor to the variability reported in both the diagnostic and prognostic performance of *R*_out_.

A related issue concerns the definition of “non-response”. Patients classified as non-responders do not necessarily constitute a homogeneous group. Some may have been incorrectly diagnosed, others may harbor substantial concomitant neurodegenerative pathology that limits postoperative improvement, and still others may have potentially shunt-responsive iNPH but experience an unfavorable outcome because of suboptimal valve settings, underdrainage, shunt-related complications, or occult shunt malfunction. Consequently, failure to demonstrate clinical improvement after surgery cannot be assumed to indicate the absence of iNPH or the absence of a physiologically relevant disturbance in CSF dynamics. The duration of follow-up and the independence of outcome assessment introduce further methodological challenges. Response to shunt surgery in iNPH may be transient and may differ across clinical domains, with cognitive improvement often lagging behind gait or functional recovery. As a result, outcome assessments performed at different time points may yield substantially different response rates. Early assessments may fail to identify delayed responders, whereas prolonged follow-up may confound treatment-related improvement with the natural progression of underlying neurodegenerative disease. Consequently, variability in follow-up duration is likely an important contributor to the heterogeneity observed across studies. Moreover, outcome assessments performed by unblinded investigators may be vulnerable to expectation and observer bias, further complicating comparisons across studies.

A patient classified as a non-responder may, in fact, have genuine iNPH and an adequately functioning shunt but may not yet have demonstrated measurable clinical improvement at the time of assessment. Conversely, the absence of improvement may reflect inadequate CSF diversion due to suboptimal valve settings, underdrainage, shunt malfunction, or other shunt-related problems rather than a true lack of treatment responsiveness. For this reason, patients initially classified as non-responders should ideally undergo systematic re-evaluation several months after surgery, including assessment of shunt function when appropriate. However, such reassessment has rarely been incorporated into published studies, where failure to improve at a predefined follow-up interval is often accepted as definitive evidence of non-response. Pooling genuinely non-responsive patients with those who have inadequate shunt drainage or occult shunt malfunction inflates the apparent non-response rate and lowers the apparent NPV of the tests used to select them for surgery.

## 11. Alternative Diagnostic Approaches to iNPH

As discussed in the 2021 Japanese Clinical Practice Guidelines and other contemporary recommendations, several ancillary investigations are available to support the diagnosis of iNPH and to improve selection of patients for surgery [4,19,24]. These include the CSF tap test, external lumbar drainage, ICP monitoring, and a variety of neuroimaging-based approaches. Although each of these techniques provides valuable diagnostic or prognostic information, none has achieved sufficient accuracy to predict shunt responsiveness when used in isolation. Consequently, current diagnostic strategies rely on the integration of clinical, radiological, and physiological information rather than on the results of any single test. The following sections briefly review the strengths and limitations of the principal alternative approaches and place the role of the LIT within this broader diagnostic framework.

### 11.1. Structural Neuroimaging and MRI-Based Markers

Structural neuroimaging plays a central role in the evaluation of iNPH and contributes to patient selection through several well-established markers, including Evans’ index as the standard radiological criterion for ventriculomegaly, measurement of the callosal angle on coronal MRI, and the imaging pattern known as disproportionately enlarged subarachnoid-space hydrocephalus (DESH), characterized by ventriculomegaly, enlarged Sylvian fissures, and relative narrowing of the high-convexity and medial subarachnoid spaces [4,84]. A distinctive feature of the Japanese diagnostic algorithm is that patients with the characteristic DESH pattern and typical gait impairment may be classified as having “probable iNPH” even in the absence of a positive CSF tap test or drainage test [4]. The diagnostic importance of DESH has been further formalized through the development of composite imaging scores, and more recently, artificial intelligence (AI) and machine-learning approaches have been applied to automate the detection of DESH-related features and quantify ventricular morphology [84]. Several studies have associated DESH with a greater likelihood of improvement after shunt surgery and a higher probability of a positive response to the CSF tap test. However, the absence of DESH does not reliably predict treatment failure, and its negative predictive value remains limited. Consequently, although DESH is an important imaging marker that supports both the diagnosis and management of iNPH, it should not be used in isolation to exclude patients from consideration for shunt surgery.

More recently, AI-based brain volumetry has been proposed as a quantitative approach to overcome some of the limitations of traditional two-dimensional imaging markers. Sahuquillo et al. argued that ventricular enlargement in older adults should be viewed as a continuum in which disturbances of CSF dynamics and neurodegenerative atrophy frequently coexist, limiting the diagnostic usefulness of binary morphometric criteria [85]. Using automated volumetric pipelines such as *vol2Brain*, AI-based brain volumetry enables reproducible three-dimensional quantification of brain structures and CSF compartments. In addition, quantitative volumetric analysis may improve the interpretation of CSF biomarkers by accounting for dilutional effects related to enlarged CSF spaces. Although these techniques remain investigational, they may provide a more objective and biologically grounded framework for patient selection and outcome prediction in iNPH.

Phase-contrast MRI measurement of aqueductal CSF flow has been investigated as a non-invasive biomarker of altered CSF dynamics in iNPH. However, the available evidence remains inconsistent. In a study of 49 patients with iNPH undergoing ventriculoperitoneal shunting, Dixon et al. found that aqueductal CSF flow rates were not significantly associated with postoperative improvement in gait, cognition, or urinary symptoms, and many patients with normal preoperative flow measurements nevertheless experienced meaningful clinical benefit [86]. In the first meta-analysis of phase-contrast MRI studies in iNPH, Whitley et al. reported that aqueductal stroke volume and peak CSF flow velocity were significantly higher in patients with iNPH than in healthy controls, supporting the concept of hyperdynamic CSF flow in iNPH [87]. However, the prognostic value of these parameters remained inconsistent across studies, and no flow metric demonstrated sufficient reliability for predicting response to shunt surgery. The authors concluded that phase-contrast MRI should not be used as a stand-alone predictor of surgical outcome, highlighting the need for standardized acquisition protocols and flow measurements to improve reproducibility across centers [87].

### 11.2. The CSF Tap Test

The CSF tap test is the simplest, most widely available, and most commonly used ancillary investigation in the diagnostic workup of iNPH. Its practical advantages, including the absence of specialized equipment, a procedure time of less than 30 min, direct assessment of clinical response following CSF removal, and a complication profile largely limited to the risks of lumbar puncture, make it an attractive first-line test. A systematic review by Mihalj et al. (2016) reported a pooled sensitivity of 58% and specificity of 75%, corresponding to an LR+ of 2.32 and an LR− of 0.56 [88]. These findings are consistent with the position adopted by the 2021 Japanese Clinical Practice Guidelines, which state that improvement of the clinical triad following the CSF tap test strongly supports the diagnosis of iNPH and predicts a favorable response to shunt surgery [4]. Conversely, the absence of improvement does not exclude iNPH, and clinicians should remain aware of the possibility of false-negative results and subsequent improvement after shunt intervention. Taken together, the pooled S, Sp and LRs indicate that the CSF tap test functions primarily as a rule-in rather than a rule-out investigation. Thus, a positive response increases diagnostic confidence and supports proceeding to shunt surgery, whereas a negative result should not be used in isolation to exclude patients from potentially beneficial treatment.

### 11.3. External Lumbar Drainage

External lumbar drainage (ELD) is generally regarded as one of the most sensitive supplementary tests for identifying patients with iNPH who are likely to benefit from shunt surgery. The procedure involves continuous lumbar CSF drainage through an intrathecal catheter, typically over several days, allowing removal of substantially larger CSF volumes than can be achieved with a single tap test. The 2021 Japanese CPGs discuss the CSF tap test and ELD within the same section but do not provide a detailed evaluation of ELD as a separate diagnostic modality [4].

Relatively few studies have specifically investigated the role of ELD in iNPH. In a prospective study of 68 patients with suspected iNPH, Mahr et al. assessed the predictive value of clinical evaluation, 72 h ELD, overnight ICP monitoring, and the LIT [89]. Patient selection for shunt surgery was largely based on a positive ELD response together with favorable subjective assessments by patients and their relatives (*n* = 33, 48.5%). This introduces a potential source of selection bias, as the test under evaluation also contributed to the decision to proceed with surgery. Within this cohort, ELD demonstrated the highest predictive value among the investigated modalities, with a positive response correctly predicting postoperative clinical improvement in 87.9% of patients [89]. Although ELD appeared more sensitive than the CSF tap test in this study, no published evidence has conclusively demonstrated that it provides superior overall predictive accuracy for identifying patients who will benefit from shunt surgery. The principal disadvantages of ELD include its invasive nature, the requirement for hospitalization and prolonged monitoring, and the risk of complications such as infection, meningitis, CSF leakage, overdrainage, and patient discomfort. Although reported rates vary across studies, meningitis has been described in approximately 4-12% of patients undergoing prolonged lumbar drainage [90,91].

### 11.4. Intracranial Pressure Monitoring

Continuous ICP monitoring has long been proposed as an ancillary investigation in patients with suspected iNPH [62,92,93,94]. Among the diagnostic tests currently used in iNPH, it is arguably the most invasive, typically requiring hospital admission, prolonged recording, specialized equipment, and experienced personnel. As a result, it is resource-intensive and associated with procedural risks, including complications related to catheter placement and infection. However, the risk of infectious complications is generally lower than that associated with ELD [90,91]. Unlike the CSF tap test or ELD, which primarily provide a binary assessment of clinical response, ICP monitoring permits quantitative characterization of multiple physiological parameters, including mean ICP, pulse amplitude, slow vasogenic waves (B waves), compensatory reserve, and indices of intracranial compliance. From a physiological perspective, it is therefore the most comprehensive method currently available for assessing disturbances of CSF dynamics. Despite these advantages, published studies have yielded inconsistent results regarding the predictive value of individual ICP-derived parameters for shunt responsiveness. Although ICP monitoring is discussed in the major contemporary clinical practice guidelines for iNPH, it occupies a relatively limited role within current diagnostic algorithms, and the available evidence has not been reviewed as extensively as that supporting the CSF tap test, ELD, or the LIT [4,19,24]. As a result, ICP monitoring has not achieved the same degree of acceptance as the CSF tap test, ELD, or lumbar infusion testing and remains confined largely to specialized centers with expertise in ICP monitoring and CSF dynamics. Nevertheless, its limited adoption should not be interpreted as evidence of limited physiological or prognostic value. On the contrary, ICP monitoring provides access to a broad range of dynamic variables that cannot be obtained from other ancillary investigations and may offer unique insights into the pathophysiology of iNPH with a relatively low and generally acceptable procedural risk. Contemporary advances in signal processing, waveform analysis, and multimodal predictive modeling may help clarify the role of ICP monitoring in the diagnostic evaluation of patients with suspected iNPH.

In a systematic review and meta-analysis of 35 studies, Thavarajasingam et al. (2021) found that ICP monitoring demonstrated the highest predictive accuracy for shunt responsiveness, followed by ELD, the LIT, and the CSF tap test [95]. Based on these findings, the authors proposed a two-tiered diagnostic strategy in which readily available first-line investigations, such as the CSF tap test and LIT, are followed by ICP monitoring or ELD in patients who remain strongly suspected of having iNPH despite negative initial test results [95].

## 12. Beyond CSF Hydrodynamics: Proteomics and Emerging Biomarkers

The frequent overlap between iNPH and neurodegenerative disease, particularly AD, means that a substantial proportion of patients meeting the clinical and radiological criteria for probable iNPH may harbor concomitant brain pathology that limits their response to shunt surgery, irrespective of their *R*_out_ value. For example, a patient with iNPH and coexisting AD pathology may exhibit ventriculomegaly and an elevated *R*_out_, reflecting impaired CSF dynamics, while cognitive symptoms are driven predominantly by the underlying neurodegenerative process and therefore remain largely unresponsive to shunt surgery.

Neuropathological studies have shown that concomitant AD and cerebrovascular pathology are common among patients meeting clinical criteria for iNPH [96]. Of particular relevance, the presence of such comorbid pathology has been associated with poorer outcomes after shunt surgery, particularly with respect to cognitive improvement. In a prospective study of 37 patients with iNPH, Hamilton et al. demonstrated that concomitant AD pathology substantially influences the response to shunt surgery. Cortical biopsies obtained at the time of shunt insertion revealed AD-related pathology in 67.6% of patients [96]. While patients with absent or mild pathology showed significant postoperative improvement, those with moderate-to-severe AD pathology exhibited worse baseline cognitive performance and derived little or no clinical benefit from shunting [96]. These findings underscore the need for biomarkers capable of identifying coexisting neurodegenerative pathology before surgery and improving prognostic stratification.

Much of the interest in CSF biomarkers in iNPH has focused on the amyloid cascade, largely as a consequence of the intense research effort directed toward AD [97]. Because AD is characterized by the accumulation of misfolded amyloid-β and tau proteins within amyloid plaques and neurofibrillary tangles, respectively, CSF biomarkers reflecting these pathological processes have become widely used for the detection of concomitant AD pathology in patients with iNPH. Several studies have identified a characteristic CSF biomarker profile in iNPH, consisting of reduced concentrations of amyloid-β peptides and soluble amyloid precursor proteins in the presence of normal or only mildly reduced tau levels. In contrast, AD is typically characterized by elevated tau concentrations and a disproportionate reduction in amyloid-β42, providing a potentially useful biochemical distinction between the two conditions [97]. Recent advances in mass spectrometry (MS)-based proteomics have enabled detailed characterization of the CSF proteome in iNPH, expanding biomarker discovery beyond the amyloid cascade and providing new insights into the underlying pathophysiology of the disease [98,99].

In a landmark proteomic study, Ying et al. (2026) applied Gene Ontology (GO) enrichment analysis to characterize the biological pathways associated with proteins altered in iNPH [98]. The authors identified 1775 proteins in ventricular CSF samples from shunted patients. Their analysis revealed enrichment of pathways related to neuroinflammation, extracellular matrix remodeling, and neurodegeneration, underscoring the complex and multifactorial pathophysiology of the disorder [98].

Emerging evidence suggests that disturbances of CSF dynamics in iNPH are accompanied by chronic neuroinflammation involving blood–brain barrier dysfunction, complement activation, and altered cytokine signaling [98,99]. Consistent with this hypothesis, Ying et al. found increased concentrations of several inflammatory proteins, including CCL2 (MCP-1), CXCL12, and CCL14, in patients with iNPH compared with elderly controls [98]. MCP-1 is a chemokine that recruits immune cells to sites of inflammation and is widely regarded as a marker of neuroinflammatory activity. In addition to inflammatory pathways, proteomic analyses identified alterations in proteins involved in extracellular matrix remodeling, suggesting changes in tissue architecture, cell adhesion, and perivascular homeostasis, as well as proteins associated with axonal injury, synaptic dysfunction, and reactive gliosis, indicating that neurodegenerative processes may contribute to symptom development and variability in response to shunt surgery [98].

The neuroinflammatory response observed in iNPH is considerably more complex than can be discussed within the scope of this review, and interested readers are referred to the recent systematic review by Kwiecień et al. [99]. In addition to evidence of neuroinflammation, proteomic studies have identified reduced concentrations of proteins involved in synaptic organization, neuronal connectivity, and axonal development, including L1CAM, APOE, and NRCAM. These findings suggest that iNPH is associated not only with altered CSF dynamics but also with varying degrees of neuronal dysfunction and neurodegenerative change [97,98,99]. Such molecular alterations may help explain the marked heterogeneity in clinical presentation and treatment response observed among patients with iNPH. Although a comprehensive discussion of candidate biomarkers is beyond the scope of this review, these findings support the concept that no single biomarker is likely to adequately capture the complexity of the disorder. Instead, future diagnostic and prognostic approaches will likely require integration of molecular biomarkers with neuroimaging, CSF hydrodynamics, physiological monitoring, and clinical variables within multimodal predictive models.

## 13. From R_out_ to Multimodal Biomarkers: Future Directions in iNPH

More than sixty years after Hakim’s original description, iNPH remains a syndrome in search of a diagnostic gold standard. What has changed is our understanding of why that standard has proven so elusive: not because infusion testing is fundamentally flawed, but because the disorder it seeks to characterize is far more biologically heterogeneous and more deeply intertwined with age-related neurodegenerative pathology than the original hydraulic model anticipated. Despite decades of research, no single physiological, imaging, or molecular marker has demonstrated sufficient accuracy to reliably identify all patients with probable iNPH who will benefit from shunt surgery. The available evidence suggests that this limitation reflects not only the shortcomings of individual tests but also the marked biological heterogeneity of the disorder.

More than two decades ago, these methodological challenges were explicitly recognized. In the 2005 International Guidelines, Marmarou et al. outlined the characteristics of an ideal diagnostic study, including enrollment of both possible and probable iNPH patients, administration of all ancillary tests irrespective of previous results, shunt surgery for all patients with acceptable surgical risk regardless of test findings, and the use of standardized outcome measures with high interobserver reliability [19,20]. Such a study has yet to be conducted, largely because of substantial ethical and practical constraints. As a result, estimates of diagnostic performance continue to rely on evidence generated under study designs that are vulnerable to bias. Under these circumstances, it is perhaps unsurprising that no single biomarker has emerged as a definitive predictor of shunt responsiveness.

### 13.1. iNPH as a Spectrum Disorder

Available evidence suggests that iNPH encompasses a spectrum of biological and clinical phenotypes rather than representing a single disease entity. At one end of the spectrum are patients with predominantly reversible hydrocephalus-related dysfunction and mechanical axonal stress who are more likely to improve after shunt surgery; at the other are patients with a greater burden of neurodegenerative pathology, including amyloid and tau abnormalities, who generally exhibit a less favorable response to shunt surgery.

An additional factor that is often overlooked is the delay between symptom onset, diagnosis, and treatment. Prolonged exposure to abnormal CSF dynamics may result in progressive neuronal and white-matter injury that becomes only partially reversible, thereby reducing the potential benefit of shunt surgery even in patients with genuine iNPH. Shunt responsiveness therefore reflects the balance between reversible CSF-dynamic disturbances and irreversible neurodegenerative or structural brain changes. This biological heterogeneity provides a plausible explanation for why no single biomarker, including *R*_out_, has demonstrated sufficient diagnostic or prognostic accuracy when used in isolation. Within this framework, the future role of *R*_out_ may need to be reconsidered.

Recent research increasingly supports the view that pre-existing neurodegenerative burden is among the strongest predictors of poor response to shunt surgery in iNPH [96,97,98,99]. These studies suggest that neither the clinical triad nor *R*_out_ derived from the LIT is sufficient, in isolation, for accurate prognostic stratification. Instead, molecular and biological biomarkers may be required to identify patients most likely to benefit from treatment. Collectively, these findings reflect a broader shift from viewing iNPH as a purely hydrodynamic disorder toward a more integrated model that incorporates molecular and cellular mechanisms, as well as the balance between reversible hydrocephalus-related dysfunction and irreversible neurodegenerative pathology.

### 13.2. The Future Role of R_out_

The question of why a central metric of CSF dynamics has struggled to reliably predict shunt outcomes has challenged clinicians and researchers for decades. Several factors may account for this persistent paradox. First, as discussed previously, *R*_out_ increases physiologically with age and, although elevated resistance to CSF outflow has long been considered a key physiological feature of iNPH, whether it represents a necessary prerequisite for the development of the syndrome remains uncertain. Many older adults exhibit elevated *R*_out_ values without displaying the clinical or radiological features of iNPH, suggesting that *R*_out_ reflects an important but not exclusive component of the underlying pathophysiology. Second, technological advances in computerized data acquisition and signal analysis now allow extraction of additional CSF dynamic parameters, particularly craniospinal compliance, that could be incorporated into the routine interpretation of the LIT. Compliance plays a critical role in determining how the intracranial system accommodates volume changes, and its impairment may be a more sensitive indicator of exhausted compensatory reserve than *R_out_* alone. Although several investigators have proposed and implemented compliance-related metrics, their use has largely remained restricted to a limited number of specialized research centers [100,101].

### 13.3. The Reference Standard Dilemma

One potential solution to the reference standard dilemma in iNPH is the use of a composite reference standard. Rather than classifying patients solely according to postoperative improvement following shunt surgery, a composite standard integrates multiple sources of information, including clinical assessment, quantitative gait measures, cognitive testing, functional outcome scales, neuroimaging findings, and potentially CSF or blood biomarkers. Patients are then classified according to the combined weight of evidence provided by these complementary measures. Such an approach may better reflect the multifactorial nature of iNPH, reduce the influence of errors associated with any single outcome measure, and provide a more accurate representation of the underlying biological and clinical complexity of the disorder.

Although composite reference standards offer a potential solution to the absence of a definitive gold standard in iNPH, their implementation is not without challenges. Fundamental questions remain regarding which variables should be included, how individual components should be weighted, and how the resulting standard should be validated. Furthermore, the marked clinical and biological heterogeneity of iNPH raises concerns about the generalizability of any single composite definition across different patient populations. Consequently, although composite reference standards may reduce some of the limitations associated with outcome-based definitions of shunt responsiveness, they also introduce new methodological complexities that require careful consideration.

### 13.4. Toward Multimodal Predictive Models

Recent machine-learning analyses of CSF proteomic profiles further support this direction by demonstrating that combinations of molecular biomarkers may improve prognostic stratification beyond the performance achievable with any single marker alone [98]. More broadly, future predictive models are likely to integrate molecular biomarkers with neuroimaging, CSF hydrodynamics, ICP-derived measures, and clinical variables. Such multimodal approaches may better capture the biological heterogeneity of iNPH and provide more accurate individualized estimates of shunt responsiveness than any single biomarker considered in isolation.

The path forward is unlikely to lie in the search for a single *R*_out_ threshold, but rather in embedding infusion testing within a standardized, multimodal, and probabilistically interpreted diagnostic framework. We hypothesize that the future of outcome prediction in iNPH will depend less on identifying a single superior biomarker and more on integrating complementary sources of information. By combining *R*_out_, quantitative neuroimaging measures, cognitive profiles, and CSF proteomic signatures within a unified predictive framework, it may become possible to estimate shunt responsiveness with substantially greater accuracy than can be achieved by any individual marker alone. Such multimodal models would better reflect the multifactorial nature of iNPH and could ultimately support more personalized patient selection and shared decision-making for shunt surgery.

### 13.5. Beyond the Responder/Non-Responder Paradigm

Clinical improvement after surgery may vary substantially across symptom domains, with gait, cognition, and urinary dysfunction often showing different magnitudes and time courses of recovery. Reducing such complex outcomes to a binary classification inevitably results in loss of information and may contribute to the modest predictive performance observed for individual biomarkers. A Bayesian framework provides a useful and contemporary alternative to traditional dichotomous classifications. Rather than categorizing patients as responders or non-responders, Bayesian approaches estimate the probability of treatment benefit by combining pre-test clinical information with LRs derived from ancillary investigations. Such approaches would be better suited to capturing the biological heterogeneity of iNPH and could generate individualized estimates of treatment response based on the combined contribution of physiological, imaging, molecular, and clinical variables. From this perspective, the more clinically relevant question is not whether a patient is a responder or non-responder, but the probability and magnitude of improvement that can reasonably be expected after shunt surgery.

To fully realize the promise of precision medicine and shared decision-making in iNPH, clinicians require accurate estimates of both pre-test and post-test probability tailored to individual patients. Advances in machine learning and AI offer the possibility of generating such estimates by integrating clinical features, neuroimaging findings, CSF hydrodynamics, cognitive profiles, and molecular biomarkers. Rather than relying on a single threshold-based test result, AI-assisted multimodal models may ultimately provide personalized estimates of shunt responsiveness, allowing diagnostic evidence to be interpreted within the biological and clinical context of each patient.

A parallel priority is the standardization of outcome definitions. Future studies should adopt predefined criteria for shunt responsiveness, standardized outcome scales, fixed follow-up intervals, and independent blinded outcome assessment. Standardization should extend beyond outcome scales to include follow-up duration and timing of assessment. Evaluations performed at predefined intervals (e.g., 6, 12, and 24 months) would help distinguish early responders, delayed responders, transient responders, and true non-responders, thereby providing a more nuanced characterization of treatment response. Greater methodological uniformity would reduce between-study heterogeneity, improve the comparability of diagnostic accuracy estimates, and facilitate more reliable evaluation of existing and emerging biomarkers.

### 13.6. Conclusions and Future Perspectives

This review makes three principal contributions to the current literature. First, it provides a comprehensive historical and physiological reappraisal of the lumbar infusion test and its principal biomarker, *R_out_*. Second, by recalculating and comparing LRs derived from guideline-supported studies and published meta-analyses, it offers a unified Bayesian interpretation of the prognostic performance of commonly used *R*_out_ thresholds and their implications for predicting shunt responsiveness. Third, it highlights the absence of a universally accepted reference standard as a fundamental methodological limitation that constrains the interpretation of all ancillary investigations in iNPH, including infusion testing, tap testing, external lumbar drainage, and physiological monitoring.

Taken together, these observations suggest that reliance on *R_out_* alone oversimplifies the complexity of CSF dynamics and the biological processes underlying iNPH. The future of iNPH research is therefore likely to depend less on the search for a single diagnostic test and more on the development of integrated physiological, imaging, and molecular models capable of capturing the heterogeneity of the disorder. A multimodal approach incorporating compliance-related metrics, pulsatility measures, neuroimaging markers, and emerging molecular biomarkers may provide a more robust and clinically meaningful assessment of shunt responsiveness. Rather than being viewed as a stand-alone predictor, *R*_out_ may ultimately find its greatest value as one component of a broader diagnostic framework that integrates complementary physiological and biological information. Such an integrated framework is likely to improve patient selection, refine prognostic stratification, and support a more personalized approach to the management of iNPH.

## Figures and Tables

**Table 1:** Simulated 2 × 2 contingency table illustrating the calculation of diagnostic accuracy metrics for an *R_out_* threshold of 12 mmHg·min/mL.

	Reference Standard					
		Shunt Responder		Non-Responder		Totals
**Diagnostic test**	**Positive (Rout > 12)**	90	a	b	10	100
	**Negative (Rout ≤ 12)**	7	c	d	23	30
	Totals	97			33	**130**

In this hypothetical cohort of 130 patients undergoing shunt surgery, 97 patients experienced postoperative improvement and 33 did not. The selected threshold yielded a sensitivity of 92.8% (90/97) and a specificity of 69.7% (23/33). Based on these values, the positive likelihood ratio (LR+) was calculated as 0.928/(1 − 0.697) = 3.1. An LR+ of 3.1 indicates that a positive test result (*R_out_* > 12 mmHg·min/mL) is approximately three times more likely in a patient who will improve after shunt surgery than in a patient who will not. This represents only a modest increase in the probability of postoperative improvement and falls well below LR+ values generally considered strong evidence for predicting treatment response. **Note:** The values presented are hypothetical and are intended solely to illustrate the calculation and interpretation of diagnostic accuracy measures and likelihood ratios. In the contingency table, a = true positives (TP), b = false positives (FP), c = false negatives (FN), and d = true negatives (TN). Green cells indicate correctly classified cases (true positives and true negatives), whereas red cells indicate incorrectly classified cases (false positives and false negatives). **Abbreviations:** *R*_out_, resistance to cerebrospinal fluid outflow; LR+, positive likelihood ratio.

**Table 2:** Reported sensitivity (S), specificity (Sp), and derived likelihood ratios for the most commonly used *R*_out_ thresholds in idiopathic normal-pressure hydrocephalus (iNPH).

Author	R_out_	S	Sp	LR(+)	LR(−)
Kim 2015 [83]	10	91.6	20.7	1.15	0.41
	12	80.3	46.8	1.51	0.42
	15	64.3	62.0	1.69	0.58
	18	42.7	75.2	1.72	0.76
van Bilsen 2025 [82]	12	76.9	34.0	1.17	0.68
	14	81.6	37.2	1.30	0.49
	18	36.6	78.0	1.66	0.81

Because postoperative improvement following shunt surgery served as the reference outcome in the underlying studies, these estimates primarily reflect the prognostic performance of *R*_out_ for predicting shunt responsiveness rather than its diagnostic accuracy for iNPH itself. Positive (LR+) and negative (LR−) likelihood ratios were calculated from the published sensitivity and specificity estimates because neither meta-analysis reported these parameters. The resulting values facilitate Bayesian interpretation of the proposed *R*_out_ thresholds and illustrate the extent to which LIT results modify the probability of postoperative improvement. Sensitivity and specificity values were reproduced from the original meta-analyses; only the corresponding LRs were derived. Confidence intervals for the derived likelihood ratios could not be calculated consistently because the published meta-analyses did not provide the necessary underlying data for all thresholds; therefore, the reported likelihood ratios should be interpreted as point estimates only. Importantly, the estimates reported by Kim et al. and van Bilsen et al. were derived from different evidence syntheses, study populations, and methodologies. Consequently, values presented in adjacent rows should not be interpreted as representing different *R*_out_ thresholds evaluated within the same patient cohort or meta-analytic dataset.

## Data Availability

No new data were created or analyzed in this study. Data sharing is not applicable to this article.

## Abbreviations

The following abbreviations are used in this manuscript:

**Abbreviation**	**Definition**
AAN	American Academy of Neurology
AD	Alzheimer’s disease
AI	Artificial intelligence
APOE	Apolipoprotein E
CCL2	C-C motif chemokine ligand 2
CSF	Cerebrospinal fluid
Cout	Conductance to cerebrospinal fluid outflow
CXCL12	C-X-C motif chemokine ligand 12
DESH	Disproportionately enlarged subarachnoid-space hydrocephalus
EBM	Evidence-based medicine
ELD	External lumbar drainage
GO	Gene Ontology
ICP	Intracranial pressure
If	Rate of cerebrospinal fluid formation
iNPH	Idiopathic normal-pressure hydrocephalus
LIT	Lumbar infusion test
LR	Likelihood ratio
LR+	Positive likelihood ratio
LR−	Negative likelihood ratio
MCP-1	Monocyte chemoattractant protein-1
MRI	Magnetic resonance imaging
MS	Mass spectrometry
NRCAM	Neuronal cell adhesion molecule
NPH	Normal-pressure hydrocephalus
NPV	Negative predictive value
PC-MRI	Phase-contrast magnetic resonance imaging
PEG	Pneumoencephalography
PPV	Positive predictive value
Pss	Superior sagittal sinus pressure
PVI	Pressure–Volume Index
Rout	Resistance to cerebrospinal fluid outflow
SSS	Superior sagittal sinus
STARD	Standards for Reporting Diagnostic Accuracy Studies
STARD-AI	STARD for Artificial Intelligence
VA	Ventriculoatrial
VAS	Ventriculoatrial shunt
